# First 3D imaging characterization of Pele’s hair from Kilauea volcano (Hawaii)

**DOI:** 10.1038/s41598-018-37983-9

**Published:** 2019-02-08

**Authors:** C. B. Cannata, R. De Rosa, P. Donato, S. Donato, G. Lanzafame, L. Mancini, B. F. Houghton

**Affiliations:** 1 E3 Soc. Coop. Startup UNICAL-Arcavacata di Rende, Cosenza, Italy; 2 DiBEST, University of Calabria, Arcavacata di Rende, Cosenza, Italy; 3 Department of Physics, University of Trieste, Trieste, Italy; 4 INFN, Sezione di Trieste, Trieste, Italy; 5 Elettra-Sincrotrone Trieste S.C.p.A., Basovizza, Trieste Italy; 6 SOEST, University of Hawai’I, Manoa, Hawaii USA

## Abstract

In this work the morphologic features of Pele’s hair formed during three different eruptions of Kilauea volcano have been investigated: fountaining from Kilauea Iki’s 1959 Episode 1, weak explosive activity from Halemaumau lava lake and littoral explosions at Waikupanaha (2009). Morphological studies were performed by optical, stereo- and scanning electron microscopy. For the first time 3D image analysis was carried out by synchrotron radiation X-ray computed microtomography, which allowed a high-resolution 3D reconstruction of the internal structure of each Pele’s hair, highlighting several differences in terms of number density, elongation and shape of the vesicles between the samples from the three eruptions. We identified three main parameters determining these differences: initial size of the magma droplet, ejection velocity and magma viscosity. Pele’s hair erupted during the Kilauea Iki’s fountaining shows the highest thickness and the least elongated shape of the vesicles, though it is related to fast ejection of a low viscosity magma. We therefore suggest that the size of magma droplets is the main parameter influencing the morphology and inner textures of the Pele’s hair. The comparison with Pele’s hair of similar eruptions elsewhere demonstrates that there is no univocal correspondence between eruptive style and Pele’s hair texture.

## Introduction

Named for the Hawaiian goddess of fire^1^, Pele’s hair is an unusual type of pyroclast with highly elongated shapes. It principally occurs in Hawaiian eruption deposits, but is produced at many other basaltic volcanoes such as Piton de Neige and Piton de La Fournaise, Etna, Stromboli and Masaya^2–6^. Typically, Pele’s hair is associated with weak subaerial explosive eruption of low-viscosity basaltic melt ^4^. Pele’s hair is frequently found in Hawaiian fountains^3^, but is also common in a range of different eruptive styles: passive outgassing of lava lakes, littoral explosions, Strombolian activity^7^, submarine activity^8,9^. Previous studies on Pele’s hair lack a description or analysis of clast morphology and inner structure^10^, show pictures of the fragments^2,8,9^, present quantitative glass chemical analysis^9^ or a qualitative description of morphology and inner structure^3,5,7^. A few studies have investigated the process of formation of the Pele’s hair experimentally^11–13^ but no attempt has been made to relate their mechanism of formation with shape and/or texture. The reason probably lies in the difficulties in investigating these very thin and fragile filaments using common optical or electronic microscopy and in obtaining reliable 3D models essential to investigate the role of the different factors governing their morphology and inner structure.

Here we present the first 3D quantification of the external morphology and of the inner structure of Pele’s hair from Kilauea volcano, the type locality for Hawaiian eruptive styles. We have sampled Pele’s hair from three different eruptive mechanisms: (1) lava fountains from the Kilauea Iki eruption of 1959; (2) on-going weak explosive activity during outgassing from the surface of the lava lake of Halemaumau; (3) littoral explosions related to the interaction between lava and seawater, when lava flows reached the ocean at Waikupanaha in 2008–10 (see Supplementary Fig. S1). Particular attention was devoted to the characterization of Pele’s hair vesicle size and shape. A number of studies have investigated the vesicle texture of pyroclasts and lavas in order to reconstruct the process of vesicles formation, expansion and coalescence before, during and after the eruption^14–17^. Applying this approach to thin strands of rapidly quenched Pele’s hair will allow to examine the processes of vesiculation and crystallization on very short time scales, from the eruption to the complete quenching of the hair. These results, though obtained from a single volcano, will have a wider validity and will be applicable at a more general scale. The identification of characteristic textural and morphological features for Pele’s hair formed by different style of eruptions will help to test if such features can be unequivocally linked to their initial formation mechanism. If this is the case, the recognition of certain shapes and textures in Pele’s hair from older eruptions could help in reconstructing the eruptive mechanisms.

We present a novel combination of 2D (optical microscopy (OM) and scanning electron microscopy (SEM) on thin sections) and 3D (synchrotron radiation computed microtomography with hard X-rays (SR-μCT) using a third generation source) methods to reconstruct the internal structure of the fragments. This non-destructive methodology is well suited for the very thin (maximum diameter ~ 900 µm) and fragile Pele’s hair.

During recent years, X-ray μCT has been increasingly applied to the morphological and textural characterization of materials including its application in geosciences^18,19^; Proussevitch and co-workers^20^ introduced new analytical techniques for the statistical analysis of vesicle populations in volcanic rocks. Recently, the combination of conventional and SR-μCT has proved to be a powerful tool to obtain high-definition reconstruction of vesicles and crystals distribution of igneous products^21–26^.

Thanks to the available energy range, the high photon flux and the geometrical features of the X-ray beam, SR-μCT allows to image different regions of large scale objects (up to the tens of centimeters in size) at high contrast and spatial resolution (down to the sub-micron scale)^23,27^. In particular, the monochromaticity, the nearly-parallel geometry and the high spatial coherence of the X-ray beam can result in reconstructed tomographic images showing reduced artifacts, enhanced contrast and higher definition with respect to conventional laboratory sources^21,28,29^. The use of a free space propagation-based phase-contrast SR-μCT technique is particularly useful to visualize thin glass-bubble interfaces when their thickness is at the limit of the spatial resolution of the employed detector system.

## Results

### Pele’s hair external morphology and composition

**Figure 1:**
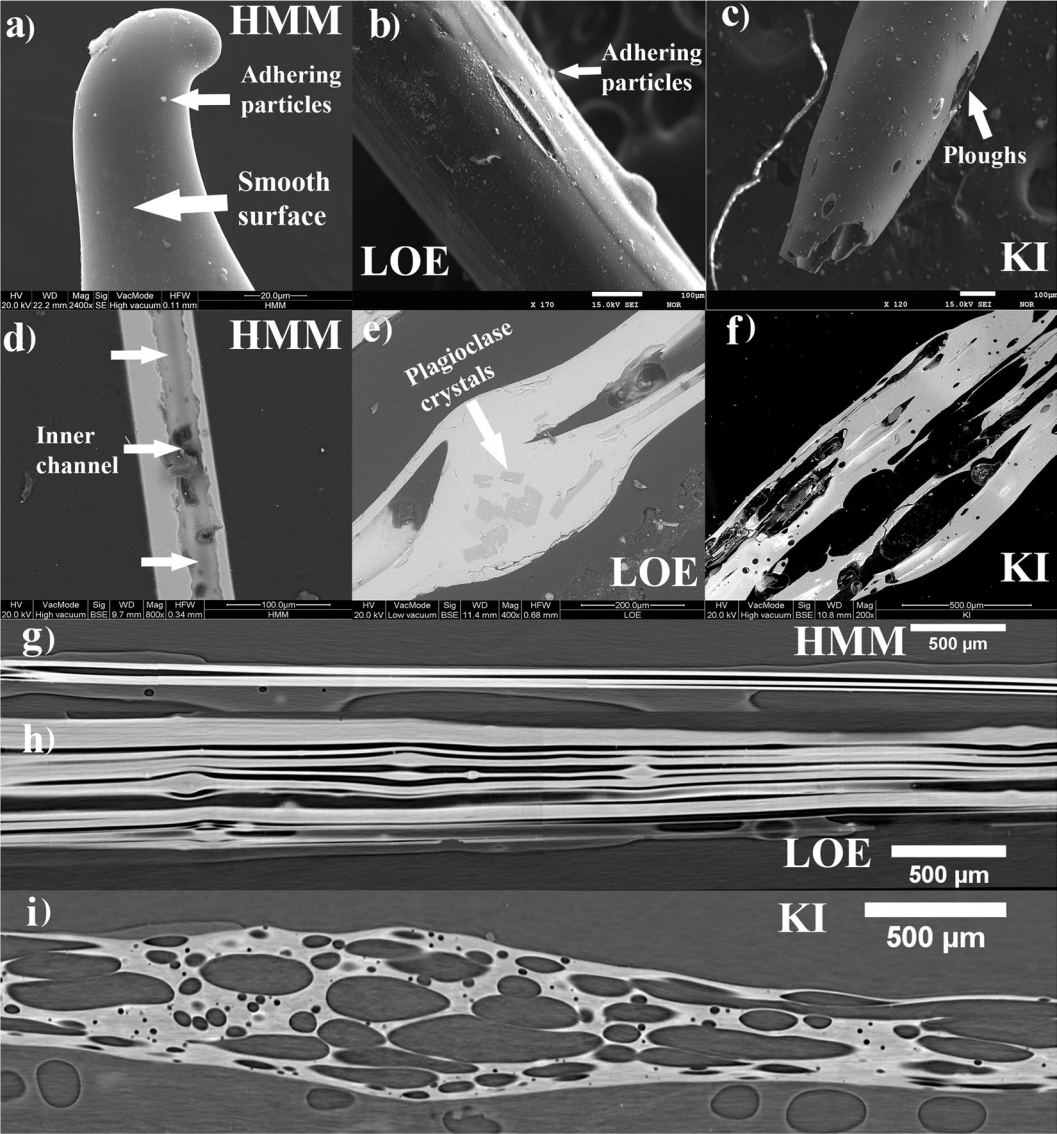
Outer and inner structure of Pele’s hair: (**a**) HMM Pele’s hair termination and adhering particles on its smooth surface - SEM SE (Secondary Electrons) image; (**b**) outer surface of LOE sample with visible adhering particles on the surface of the fragment - SEM SE image; (**c**) image outer surface of KI sample with plagues on the surface of the fragment - SEM-SE image; (**d**) empty channel along the axial direction of the hair in the thin section of HMM sample - SEM Back-Scattered Electrons (BSE) image; (**e**) thin section of LOE sample - SEM BSE image; (**f**) thin section of KI sample with the two population of vesicles visible in the picture - SEM BSE; (**g**) SR-μCT sagittal slice of HMM sample showing the presence of a channel that extend along the full length of the hair; (**h**) SR-μCT sagittal slice of LOE sample showing the presence of many elongated and few rounded vesicles, sometimes bent due to the presence of microlites; (**i**) SR-μCT sagittal slice of KI sample clearly showing the presence of different type of vesicles: small and almost round or large and elongated.

**Figure 2:**
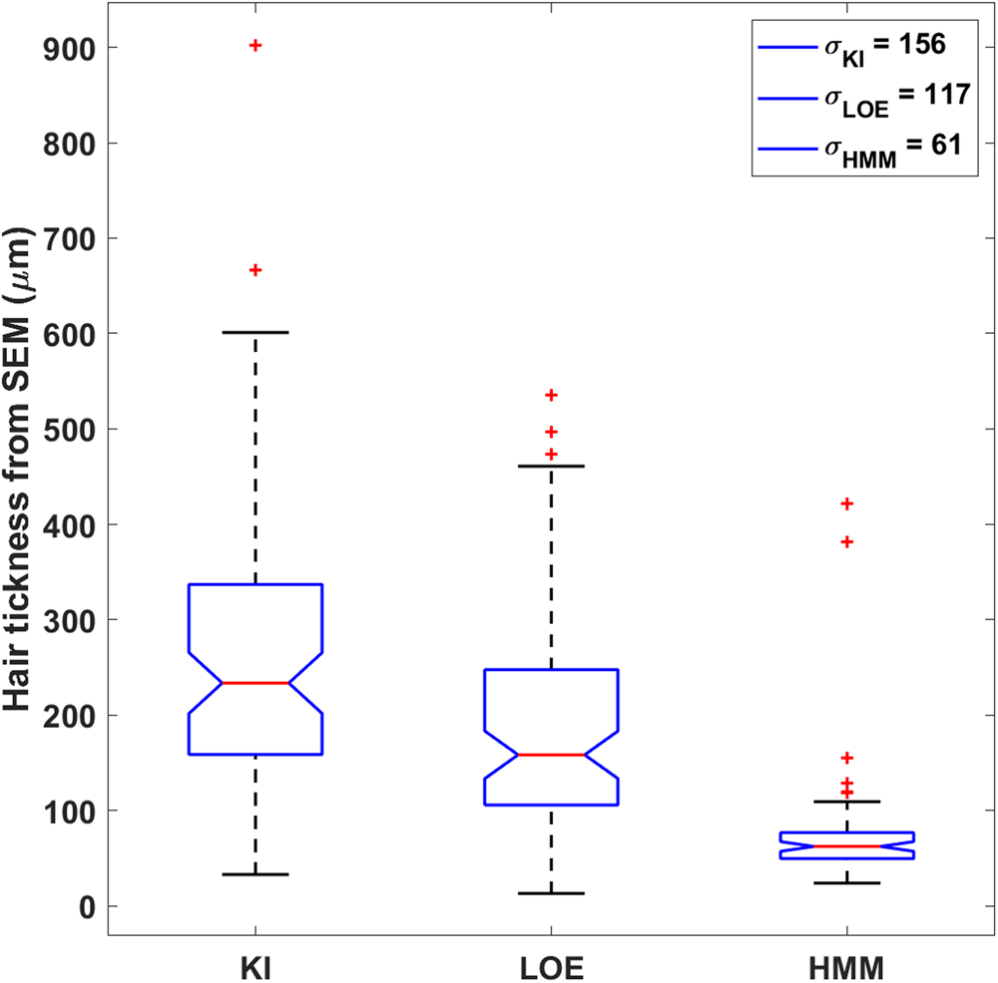
Hair thickness differences. Boxplot for the hair thickness measured by SEM on ~80 samples for each eruption type specimen. The central red line indicates the median, and the bottom and top edges of the box indicate the 25^th^ and 75^th^ percentiles, respectively. Triangular markers display the confidence interval around the median. The whiskers extend to the most extreme data points not considered outliers. KI samples are the thickest, HMM the thinnest. LOEs values place in between the previous two. The notch (triangular markers) do not overlap, indicating that the distribution medians strongly differ. Median values are: 233, 158 and 62 µm respectively for KI, LOE and HMM samples.

At hand-specimen scale, Pele’s hair resembles long strands of golden hair of variable length, from 0.5 to hundreds of millimeters. They easily snap and fragments are often incomplete. Observation under the binocular microscope and SEM revealed that the samples collected from the three different eruptions show different shapes (Fig. 1). Pele’s hair samples from Halemaumau lava lake activity (HMM) are the most elongated and closely similar to human hair, typically ranging from 50 to 100 µm in thickness (Supplementary Table S1). In contrast, the Kilauea Iki samples (KI) are the shortest and their thickness is generally of several hundreds of µm, reaching also 900 µm (Supplementary Table S1). Pele’s hair from littoral explosion (LOE) is intermediate between KI and HMM, with both thin hairs, less than 100 µm thick, and coarser pyroclasts with thickness up to 500 µm (Supplementary Table S1). The difference in the thickness of the Pele’s hair is also evident in the box plot of Fig. 2 (median values are: 233, 158 and 62 µm respectively for KI, LOE and HMM samples, and confirmed with a one-way ANOVA test, n_KI_ = 77, n_LOE_ = 80, n_HMM_ = 71, p-value < 0.0001).

The outer surface of Pele’s hair is mostly smooth (Fig. 1a) but can be rough, with ‘ploughs’ (Fig. 1c) and also large cavities. Pele’s hair from HMM shows smooth surfaces on which numerous adhering particles occur (Fig. 1a). Products from fountaining KI activity have rough surfaces with small ploughs (Fig. 1c). Pele’s hair from LOE activity shows textural features intermediate between HMM and KI. It has shapes with smooth surfaces, covered by adhering particles (Fig. 1b).

LOE and HMM glasses have similar SiO_2_ contents (ca 52 wt%), but HMM is slightly more MgO-rich; KI glass shows more primitive composition, with lower silica (ca 50 wt%) and higher MgO (ca 10 wt%) (Supplementary Table S2).

### Pele’s hair inner structure

Internal features of Pele’s hair were investigated in 2D by OM and SEM in thin sections parallel to the major axes. Microscopic observation revealed the presence of vesicles inside all the analyzed hairs. However, the shape and distribution of vesicles are different. In KI samples we observed two main populations of vesicles: one larger (major axis from 1 to 3 mm, minor axis from 0.1 to 0.5 mm), elongated parallel to the main axis of the hair, the other smaller (diameters ranging from less than 10 µm to 150 µm) and sub-rounded, dispersed across the hair (Fig. 1f). Pele’s hair from HMM contains always very elongated vesicles forming empty inner channels (Fig. 1d). LOE Pele’s hair has highly elongated vesicles, but not always extending over the entire length of the fragment, and a few smaller rounded vesicles.

Although HMM Pele’s hair is completely aphyric, samples from KI and LOE contain rare microlites of olivine (Fo_83–85_) and plagioclase (An_71–73_), respectively. When present, olivines in KI represent less than 1 vol% and reach a maximum size of 200 µm, sometimes showing evidence of skeletal growth. The abundance of plagioclase in the few crystalline LOE fragments is estimated around 5 vol% with a maximum size of 100 µm (Fig. 1e).

**Figure 3:**
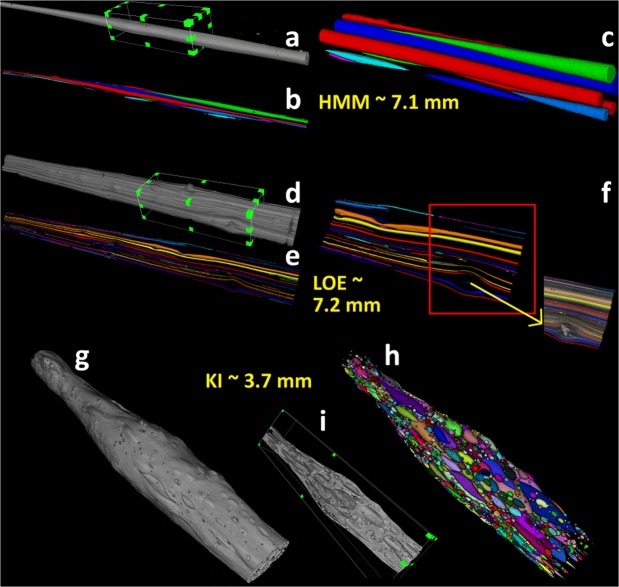
3D rendering of selected samples from HMM, LOE and KI (isotropic voxel size of 1.3 × 1.3 × 1.3 µm^3^); (**a, d**) 3D rendering of the imaged Pele’s hairs in grayscale for HMM (sample HMM5) and LOE (sample LOE2) respectively; (**b, e**) the corresponding labeled vesicles, displayed with an RGB colormap. It can be noticed that vesicles have very elongated structure and can cover the total length of the hairs; (**c, f**) enlargement of the labeled vesicles, selected from a region of interest (marked with a box) on the grayscale 3D images, for HMM and LOE samples respectively. The small insert in (**f**) shows an overlay between vesicles and hair in a subregion of the LOE sample. It shows that the bending of the vesicles is associated to the presence of small microlites; (**g**) 3D rendering of a KI3 sample and its segmented vesicle (**h**) showing a high vesicularity and two main population of vesicles: (i) round and small or (ii) large and elongated; (**i**) cut of the volume along the sagittal direction (not in scale). Length of full imaged hair are also reported in yellow text.

A detailed study of the inner structure of the samples was performed by qualitative and quantitative analysis on the 3D images obtained by SR-μCT. 3D reconstructions of selected samples from KI, HMM and LOE are shown in Fig. 3 and confirm that HMM Pele’s hair contains long vesicles running over the full length of the fragment (sagittal section: Fig. 1g; rendering: Fig. 3a–c), while several, more discontinuous channels occur inside LOE Pele’s hair (sagittal section: Fig. 1h; rendering: Fig. 3d–f). Vesicles of LOE samples are sometimes bent (see Fig. 3e) in correspondence of small microlites (Fig. 3f). Vesicle diameters are in the range of 4–130 μm and 3–188 μm for HMM and LOE samples, respectively. Measured vesicle lengths are in the range 7–7800 μm for HMM and 10–9600 μm for LOE samples. SR-μCT images of KI samples (sagittal section: Fig. 1i; rendering: Fig. 3g–i) clearly show higher vesicularity and a wide range of vesicle sizes (details in the next section). These values are comparable to those observed in 2D. Additional 3D rendering are reported in Supplementary Information as Supplementary Figs S2–S4 for HMM, LOE and KI samples, respectively.

**Figure 4:**
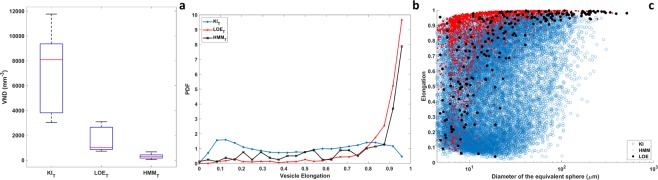
Eruption type comparison. (**a**) Boxplot of the Vesicle Number Density (VND) per melt volume for each eruption type, computed dividing the number of observed vesicles by the volume of the melt phase of each sample. The central red line indicates the median, and the bottom and top edges of the box indicate the 25^th^ and 75^th^ percentiles, respectively. The whiskers extend to the most extreme data points not considered outliers. VND for KI samples shows the highest values, while for HMM the lowest. VND for LOE are in between the previous two; (**b**) show the Probability Density Function (PDF) computed from the elongation of all the segmented vesicles grouped by eruption type. LOE and HMM samples show a very elongated structure, while PDF for KI samples is almost flat indicating the presence of a mixture of round and very elongated objects (significant differences checked with a one-way Anova test, n_KI_ = 17500, n_LOE_ = 1100, n_HMM_ = 200, p-value < 10^−5^). Bins are equally spaced in the range determined by the minimum and maximum measured values between the three groups; (**c**) scatterplot of the elongation as a function of the equivalent spherical diameter of the vesicle. In HMM and LOE Pele’s hair most of the vesicles are strongly stretched. Only few, small and rounded bubbles occur in these two kinds of fragments. The correlation between elongation and size is less evident in KI vesicles.

The most relevant parameters obtained by the 3D quantitative analysis of vesicles are summarized in Table 1 and shown in Fig. 4 (detail of the statistical analysis in section 4.4). On average, KI Pele’s hair displays higher vesicularity (as percentage of volume occupied by vesicles) than samples from the other two groups. Moreover, KI samples also show a significantly higher Vesicle Number Density (VND) per melt volume (Fig. 4a, median value: 8082 mm^−3^); VND furtherly decreases from LOE to HMM (median values: 1005 and 291 mm^−3^, respectively). VNDs are statistically different (Mann-Whitney-Wilcoxon U test, n_KI_ = 9, n_LOE_ = 8, n_HMM_ = 13, p-value < 0.0001). A parameter strongly discriminating the KI samples from the other two groups is the elongation of the vesicles. Figure 4b shows the probability density function (PDF) of vesicle elongation. While HMM and LOE display similar patterns, with a maximum PDF for highly elongated vesicles, KI has a rather “flat” pattern, indicating that both highly and poorly elongated vesicles are common. The scatterplot of the elongation as a function of the diameter of the equivalent sphere (Fig. 4c) highlights that in HMM and LOE Pele’s hair most vesicles are strongly stretched, regardless their dimensions. Only few, small and rounded vesicles occur in these two kinds of fragments. On the contrary, in KI vesicles the correlation between elongation and size is less evident, and rounded vesicles are abundant in almost all the size classes.

**Table 1:** Sample list and results of quantitative analysis described in section 4.3.

	V_hair_ (mm^3^)	L_hair_ (mm)	D_hair_ (mm)	Vesicularity (vol.%)	N	*l* (mm)	Deq (μm)	Vesicles average elongation	N’ (%)	VND (mm^-3^)
**LOE SAMPLES**										
LOE_1	0.54	7.20	0.31	16.7	367	0.6	14.8	0.91	0.8	812.6
LOE_2	0.27	7.23	0.26	10.3	249	0.5	13.2	0.78	1.6	1018
LOE_3	0.04	7.18	0.10	6.2	31	1.3	7.9	0.96	6.5	897.1
LOE_4	0.04	7.19	0.11	8.6	108	0.5	8.5	0.93	2	3085.5
LOE_5	0.14	9.47	0.15	23.1	107	0.7	16.3	0.87	1.9	992.3
LOE_6	0.08	9.38	0.10	8.9	186	0.6	7.9	0.92	1.1	2590.1
LOE_7	0.11	9.43	0.14	1.5	75	0.1	12.4	0.74	0	713.3
LOE_8	0.03	8.13	0.09	9.4	77	0.8	7.1	0.87	1.3	2691.3
**KI SAMPLES**										
KI_1	0.66	4.85	0.42	20.1	2245	0.09	20.9	0.69	0.04	3970.9
KI_2	0.25	4.48	0.27	23.1	1861	0.14	12.9	0.76	0.1	8915.8
KI_3	0.63	3.73	0.45	48.7	3010	0.06	34.5	0.31	0	9051.8
KI_4	0.45	4.58	0.34	39.6	2300	0.11	24.9	0.51	0	8082.5
KI_5	0.15	4.06	0.22	27.7	442	0.27	16.8	0.68	0	3304.4
KI_6	0.65	4.31	0.44	37.1	4388	0.06	30.3	0.32	0	10259.8
KI_7	0.34	2.82	0.39	27.1	812	0.11	23.1	0.63	1.2	3026.1
KI_8	0.16	3.65	0.23	23.2	1513	0.12	11.9	0.70	0.1	11749.1
KI_9	0.51	4.71	0.36	17.2	2241	0.11	15.9	0.61	0	5029.1
**HMM SAMPLES**										
HMM_1	0.31	7.18	0.23	35.6	47	0.69	54.3	0.83	4.3	233.7
HMM_2	0.28	7.09	0.21	18.6	39	1.05	31.9	0.84	5.1	167.3
HMM_3_A	0.03	1.61	0.16	38.6	13	0.98	26.9	0.95	38.5	661.7
HMM_3_B	0.05	5.56	0.10	22.0	12	2.56	18.4	0.97	33.3	334.6
HMM_4	0.04	7.59	0.08	32.9	8	2.65	24.1	0.81	7.7	314.6
HMM_5	0.18	7.20	0.17	20.9	65	0.52	31.3	0.82	1.5	468.2
HMM_6_A	0.07	5.07	0.13	20.8	13	2.06	22.7	0.96	23.1	220.3
HMM_6_B	0.01	1.78	0.07	10.1	3	1.77	11.6	0.99	100	543.1
HMM_7	0.06	7.06	0.10	5.6	8	0.82	22.1	0.93	12.5	149.7
HMM_8	0.04	6.02	0.09	20.4	10	2.41	17.6	0.86	40	291.5
HMM_9	0.02	5.80	0.07	18.6	8	5.07	10.9	0.95	87.5	405.6
HMM_10	0.04	7.06	0.09	2.6	2	7.06	9.2	0.99	100	45.9
HMM_11	0.04	7.03	0.08	8.4	5	2.23	19.2	0.97	20	126.3

The first three columns report the measured values of Pele’s hair volume (*V*_hair_), length (*L*_hair_) and thickness (*D*_hair_), respectively. The third column is the hair *vesicularity*, evaluated as the fraction of vesicle volume over the total volume of the sample expressed in percentage. *N* is the number of separated vesicles found in each sample. Quantified average length (*l*) and width (Deq) of the vesicles for each sample are reported in fifth and sixth column. The last two columns represent the percentage of vesicles long as the sample (N’) and the vesicle number density per melt volume (VND), respectively. VND is calculated as N/(V* (1-Vesicularity/100)).

A and B refer to different parts of a broken Pele’s hair sample.

### Pele’s hair vesicles morphology

Vesicle Size Distributions (VSD) and vesicle shape description were obtained only for Pele’s hair from Kilauea Iki samples, as they show a high VND. All analysis details and sample selection criteria are reported in section 4.4. The full dataset of vesicles from KI samples (i.e. grouping all the observation together) was used to characterize VSDs in terms of volume, length and width. Results are reported in Table 2 and the histograms of the Log10-based conversion of data are presented in Supplementary Fig. S5. VSDs are spread over several order of magnitude. Vesicle volumes for KI range from 10^2^ μm^3^ to 10^7^ μm^3^, while their widths are in the range 4–250 μm and the lengths are in the range 7–3000 μm. The VSDs give no information on the geometry of the vesicles observed in 2D and 3D images.

**Table 2:** Estimated statistical parameters from vesicle size distribution (VSD) analysis described in section 4.4.

	Geometric mean of the VSD (µm)	Geometric Standard Deviation σ*	68.7% confidence interval
KI Vesicle width Deq*	15.1	2.0	[7.7, 30.3] µm
	Geometric mean of the VSD (µm^3^)	Standard Deviation σ*	68.7% confidence interval
KI Vesicle volume V*	2.7 * 10^3^	9.7	[0.3, 26.4]* 10^3^ µm^3^
	Geometric mean of the VSD (µm)	Standard Deviation σ*	68.7% confidence interval
KI Vesicle length l*	41.7	3.0	[13.8, 125.9] µm

Grouping all the vesicles from Kilauea Iki samples, it is possible to evaluate average vesicle width (Deq*), volume (V*) and length (l*). Average values represent the geometric mean of the right-skewed distribution computed from the mean of the Log10-based transformation of the data, while σ* is the multiplicative standard deviation. The 68.7% confidence interval is reported in last column and it is correctly asymmetric due to the high skewness of data.

**Figure 5:**
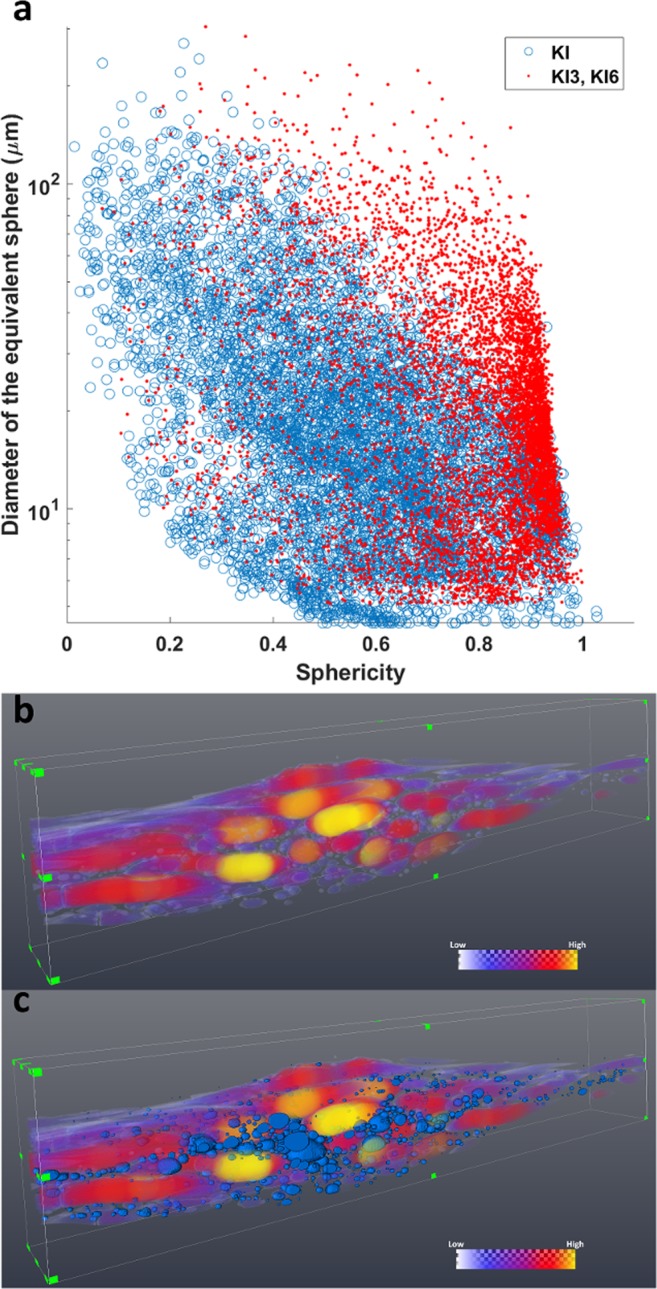
Kilauea Iki morphology. (**a**) Scatterplot of the diameter of the equivalent sphere (obtained from the volume) as function of the vesicle sphericity. Red markers indicate KI3 and KI6 samples, blue markers all the others. The majority of the samples shows a clear relationship between the size of vesicles and their shape, with the exception of KI3 and KI6, where spherical vesicles show higher size compared to other samples of the same eruption type. This is linked to a swelling of the hair that is also followed by a local increase of the thickness of vesicles. (**b**) 3D rendering showing a sagittal cut of the vesicles thickness map displayed using a color code associated to the diameter of the largest ball inscribed in the vesicle: yellow colors correspond to the higher vesicle thickness (the same image for KI6 is reported in Supplementary Fig. S6). (**c**) 3D rendering of vesicles having sphericity > 0.9 (blue objects) overlaid to the thickness map. Larger spherical vesicles are located in the swelled section of the hair.

The sphericity of vesicles was used as shape descriptor to delineate a relationship with their size. In Fig. 5a the diameter of the equivalent sphere (computed from the volume) of each vesicle is displayed as a function of the corresponding sphericity. In general, high values of sphericity correspond to small vesicles, while large vesicles show low sphericity (blue markers in Fig. 5a). Only samples KI3 (Fig. 3g) and KI6 (Supplementary Fig. 4c), characterized by a strong variation in the hair section, show large spherical-like vesicles (red markers in Fig. 5a). The 3D thickness map of the vesicles for KI3 (Fig. 5b, Supplementary Video) displays a general increase of vesicles width in correspondence of the hair swelling. In Fig. 5c the 3D rendering of vesicles having sphericity > 0.9 is overlaid to the thickness map. Notably, the coarse and elongated vesicles are mostly distributed in the inner and thickest part of the hair. The same features were also seen in sample KI6 (see Supplementary Fig. S6).

## Discussion

HMM Pele’s hair, formed during passive outgassing from a lava lake, are very long and thin. They show few, highly elongated vesicles, often extending along the entire hair, and almost no round vesicles. KI samples are related to a lava fountain and are shorter and coarser. They are more vesicular than HMM and show a higher VND. Frequently two populations of vesicles occur: a large population of elongated vesicles and a small population of mainly near-spherical vesicles (Fig. 5). Finally, LOE Pele’s hair, related to a littoral explosion, shows intermediate sizes between KI and HMM with abundant highly elongated and few smaller and rounded vesicles.

The ‘spurting’ velocity, that is the velocity of the magma droplet (Pele’s tear) when ejected into the atmosphere, is considered as the main factor controlling the formation of Pele’s hair^11^. No experimental study has been carried out on the factors governing the shape of the Pele’s hair, but it seems reasonable that higher velocities favor the formation of longer and thinner hair. Additional parameters can modulate the shape of the Pele’s hair: i) size of the original magma droplet, in turn influencing the quenching rate; ii) viscosity of the magma and iii) the residence time of the hair in the hot environment. For a lava fountain of Kilauea Iki 1959, Wilson^30^ calculated a spurting velocity of 297 m/s for the smaller pyroclasts (0.84 mm), while Mattox and Mangan^10^ calculated a minimum value of ejection velocities ranging between 1–15 m/s for a littoral explosion of Kilauea volcano. No published data about the Halemaumau lava lake is available, but for a similar lava lake at Villarrica initial velocities of 12 m/s have been calculated^31^.

The formation of Pele’s hair in viscous melts occurs when quenching time is shorter than the time of separation of the filament from the droplet to which they are attached (Pele’s tear) and the diameter of the thin strands is about 1/5 of that of the Pele’s tear^12^. According to this, we have roughly estimated the diameter of the original magma droplet considering its diameter as being 5 times the median value of the thickness distribution for each group of Pele’s hair (Fig. 2). Results show larger volumes for KI (about 1 mm^3^) than for LOE (0.2–0.3 mm^3^) and HMM (about 0.01 mm^3^). It is obvious that for the very thin HMM Pele’s hair the quench time (t_quench_) is shorter than for the thicker KI Pele’s hair. Longer t_quench_ allows the formation of a second population of vesicles not deformed by the stretching. Therefore, in Pele’s hair containing round vesicles the t_quench_ must be longer than the time of the second vesiculation (t_ves_). As shown in Fig. 4c, the rare rounded vesicles (elongation < 0.1) have a maximum diameter of the equivalent sphere of 8 µm in HMM; 20 µm in LOE and up to 100 µm in KI. With these values it is possible to calculate the maximum t_ves_ considering a vesicles growth rate of 3.2 × 10^–4^ cm/s, as reported by Mangan and co-workers^32^ for the basalts of Kilauea volcano. The results are of about 1 s for HMM, 3 s for LOE, 15 s for KI. Maximum t_quench_ are equal or higher than these values and the obtained results are consistent with the experimental finding of Mastin and co-workers^13^.

Viscosity strongly influences the capacity of a droplet to deform under the action of the gas expansion in the bursting bubbles or wind and is a function of several parameters, including the melt composition, temperature, vesicle and crystal contents^33–35^. Following Giordano and co-workers^36^ we have calculated the viscosity (η) of the three magmas, given the melt composition listed in Supplementary Table S2, assuming an average water content of 0.5 wt.%^37^ and the liquidus temperature at atmospheric pressure calculated by MELTS software^38^. For the three magmas results are η_HMM_ = 1.67, η_KI_ = 1.19 and η_LOE_ = 1.62 log Pa s (Supplementary Table S3). The influence of the pre-eruptive crystals on bulk viscosity can be considered negligible: the few microlites of olivine in KI and of plagioclase in LOE probably formed after fragmentation, as witnessed by i) their small size, ii) the presence of skeletal crystals indicating a very rapid crystallization, and iii) the distortion of vesicles clearly due to successive crystal growth (Fig. 3f). In any case their abundance is very low and most of the hair is completely aphyric. As suggested by Manga and co-workers^16^ the viscosity is strongly influenced by the shape and orientation of the vesicles: highly elongated vesicles are aligned to the flow direction and contribute less shear stress to the magma when compared to spherical vesicles. The occurrence of more elongated vesicles in HMM and LOE than in KI should support higher shearing. However, as previously shown, the t_quench_ is very fast in all three cases and the difference in the elongation of the vesicles has low influence on the viscosity.

**Figure 6:**
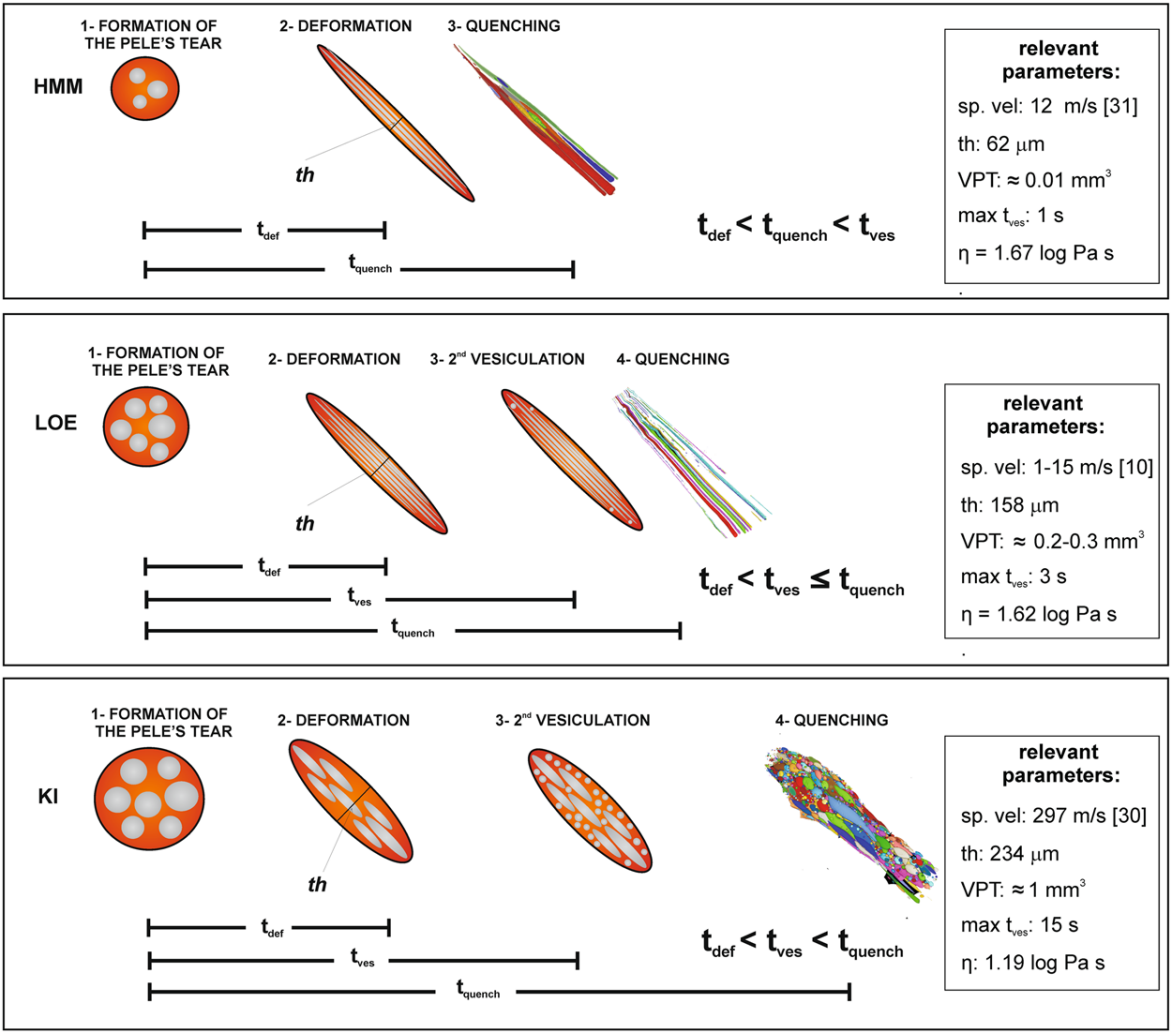
Schematic process of formation of Pele’s hair in the three studied eruptions. *t*_*def*_: time of deformation; *t*_*ves*_: time of second vesiculation; *t*_*quench*_: time of quenching. Relevant parameters discussed in the text are also reported: *sp. vel*: spurting velocity; *th*: thickness of the Pele’s hair, corresponding to the median of the thickness distribution (Fig. 2 and Supplementary Table S1). *VPT*: volume of the initial Pele’s tear, estimated considering *th* equal to about 1/5 the diameter of the Pele’s tear; *max t*_*ves*_: maximum time of second vesiculation, considering a growth rate of 3.2 × 10^-4^ cm/s and the maximum size of rounded vesicles for each sample; *η*: viscosity calculated from the chemical composition (see the text for details). For HMM the stretching of a small sized, vesiculated Pele’s tear creates a very thin Pele’s hair with long and thin vesicles. The maximum *t*_*ves*_ is in the order of 1 second; the almost complete absence of second, round vesicles indicates that the *t*_*quench*_ is shorter than this value. In LOE the coarser size of the magma droplet and the lower spurting velocity form thicker Pele’s hair, allowing, after the deformation, the nucleation of few, rounded bubbles in a maximum *t*_*ves*_ of 3 s. Their occurrence only in some fragments suggests that *t*_*quench*_ is similar to this value. In KI the coarse dimension of the original droplet is probably the main factor controlling its limited stretching, in spite of the lower viscosity of the magma and the higher spurting velocity. The relatively high thickness of the resulting Pele’s hair and the longer permanence in the hot lava fountain allow the nucleation of a second population of sub-spherical bubbles during or after the deformation. The maximum *t*_*ves*_ is in the order of 15 s and the hair quenches after this second vesiculation event.

The process of Pele’s hair formation is summarized in Fig. 6. The very elongated shape of the HMM Pele’s hair can be attributed to a combined effect of very small size of droplets formed during the rupture of bubbles enclosed within a very thin magma envelope on the surface of the lava lake, and high spurting velocity. The wind transporting the Pele’s hair away from the lava lake can also act as a strain factor. The highly elongated shape of the vesicles suggests that they were already present in the magma forming the wall of the bubbles and deformed during bursting, detachment and transport. t_quench_ is very short due not only to the small size of the magma droplet, but also to the brief residence of the fragments in a hot environment and to wind chill. Further vesiculation and crystallization is therefore inhibited and only rarely few round vesicles form after the deformation. This suggests that deformation time (t_def_) is shorter than t_quench_, allowing the pronounced stretching of the hair and vesicles. In most of the cases t_quench_ is shorter than t_ves_ and crystallization time.

The thickness of the Pele’s hair formed after the littoral explosion (LOE) is higher than the HMM case, as a consequence of low spurting velocity and of the larger size of the droplets. This is probably due to the different style of fragmentation, related to the rapid vaporization of trapped water in contact with hot magma. In these fragments the vesicles are strongly elongated in the same direction of the hair, indicating that the fragmentation occurred in an already vesiculated magma, whose vesicles rapidly deformed as the magma droplet was stretched. t_quench_ is higher than the previous case, as a consequence of the coarser diameter of the magma droplets and longer residence in the hot steam column (335–550 °C^10^). It is noteworthy that a second population of few and rounded vesicles only occurs in the thicker filaments which cooled sufficiently slowly to allow ongoing gas exsolution. Their formation and the crystallization of the few plagioclase microlites can be explained with a further, short episode of vesicle and crystal nucleation after the deformation of the droplet and before quenching.

The stubby shape of KI Pele’s hair is apparently in contrast with the viscosity calculated for this poorly evolved magma, lower than the HMM and LOE cases, and with the much higher spurting velocities. The limited elongation of the KI Pele’s hair can be explained with a less efficient stretching, probably related to the dimensions of fragments in the fountain which were coarser than at HMM or LOE. Although the spurting velocities of magma fragments in the lava fountain were very high, the higher diameter of droplets led to the formation of thicker and less elongated filaments which cool down relatively slow also because of the very long residence in the hot fountain. The occurrence of different size populations of vesicles is generally related to distinct pulses of nucleation and growth^17,27,39^. Deformation of vesicles is expressed by the Capillary number (Ca) which is directly proportional to radius^16^. Therefore, small vesicles, formed during a second pulse of nucleation, are less deformed both due to their radius and to their formation during the last stages or after the stretching, but before the quenching (t_def_ < t_ves_ < t_quench_). Coarser elongated vesicles formed before or during the stretching. This is also supported by the distribution of smaller and round vesicles across the hair cross section, while the coarser and elongated vesicles are mainly concentrated in the axial portion (Fig. 5b, c). A similar distribution of vesicles of different size and a comparable VND is observed in coarse scoria fragments of the same eruption^40^ and is interpreted as an evidence of no post-fragmentation gas expansion. However, the coarser scoria clasts are dominated by round, relaxed vesicles. In Pele’s hair the t_quench_ is short enough to prevent the elongated vesicles from relaxing to spheres^13^. Only in Pele’s hair showing evident thickening (e.g. KI3 and KI6), also the coarser vesicles show relatively higher sphericity (Fig. 5a), implying some relaxation. As in the previous case, the olivine microlites formed during or after the eruption and before the complete quenching of the hair. SR-µCT analyses revealed that KI Pele’s hair is more vesicular than the other samples. This can be related to secondary vesiculation, but is probably mainly due to the original gas content of the magma at the moment of Pele’s hair formation.

The different morphologies and vesicularities of Pele’s hair from the three eruptive styles suggest that the eruptive mechanism is a main factor determining their internal and external morphological features. However, Pele’s hair sampled during persistent degassing of Masaya volcano (Nicaragua), has coarser diameters (over 200 μm) and has both elongated vesicles extending discontinuously along the length of the hair and smaller and rounded vesicles^5^. Masaya Pele’s hair is therefore more similar to LOE or KI samples than to HMM, although the HMM samples come from a more similar eruptive style. The differences in morphology and vesiculation of Pele’s hair formed by similar fragmentation mechanisms are therefore probably related to the higher viscosity of Masaya magma. Higher viscosity melts would lead to the formation of coarser droplets stretched in thicker filaments by the gas expansion and/or by the wind. These would quench less rapidly than in the HMM case. A second episode of vesiculation could therefore occur before the complete solidification of the fragment, forming the small and rounded vesicles very rare in the HMM samples. Pele’s hair produced during littoral explosions were commonly found during the eruption of Kilauea, in the period 1992–1994^10^, and submarine hydrovolcanic explosions on Loihi seamount^8^ and Gorda Ridge^10^. In the latter two a few photographs of Pele’s hair closely resemble LOE samples, even if in^10^ fragments seem to be more irregular and thick. Anyway no description on morphologies and vesicle textures is presented and therefore it is not possible to compare them. Pele’s hair of KI (Kilauea Iki 1959) was compared with that of the 1969–72 eruption^3^, and while numerical data on the length and thickness of the Pele’s hair or vesicle dimensions are not available, the images show long and thin strands, very different from the stubby shape of KI. In the 1969–72 eruption high fountaining was restricted to episodes in 1969 and in the later years magma was frequently ponded and/or drained back into the eruptive craters suggesting a scenario more closely resembling the current Halemaumau eruption. Pele’s hair erupted during lava fountain at Piton de La Fournaise^41^ displays the occurrence of highly stretched vesicles and a second population of rounded vesicles very similar to both KI and LOE, but a much higher microlite content, probably determining a higher magma viscosity.

This study of Pele’s hair from different eruptive styles at Kilauea and qualitative comparison with hair formed from similar eruptions at other volcanoes shows that, although the fragmentation mechanism (and therefore the eruptive style) is a key parameter in controlling the shape and vesicularity of Pele’s hair, other factors are also important. Caution should be used when trying to infer the eruptive mechanism from the texture and shape of Pele’s hair. The following parameters are also important:

1. dimensions of the magma droplet originating the Pele’s hair, in its turn influencing the quenching rate;
2. spurting velocity;
3. viscosity of the magma;
4. vesicle content in the initial magma droplet;
5. residence time in the hot environment;
6. wind.

It was not possible to quantify the last three parameters. However, our data allowed to determine that dimensions of the original Pele’s tear increases from HMM to LOE and KI. Spurting velocity is much higher for KI than for HMM and LOE, while viscosity is comparable for HMM and LOE, but lower for KI. Therefore, we suggest that spurting velocity and viscosity are secondary controls, and the size of the original magma droplet is the key factor controlling the shape and texture of Pele’s hair.

The results of this study prove the capability of SR-μCT for the investigation of small and delicate volcanic products such as Pele’s hair and that the analytical protocol described in this paper can be applied to other magmas to better constrain the Pele’s hair formation process for a wider range of magma compositions and viscosities.

## Methods

### Sampling

Pele’s hair was collected in June 2010 from the three locations listed above (Supplementary Fig. S1). Pele’s hair from HMM was found dispersed into a wide area around the vent. Huge fields of Pele’s hair, sometimes forming a carpets of few tens of meter across and accumulating before an obstacle, as for example sidewalks and rocks, are ubiquitous. We sampled Pele’s hair at about 500 m from the edge of the lava lake. Pele’s hair from Kilauea Iki 1959 eruption was picked up from the bottom layer (episode 1) of the study site KI-07-07 reported in^40^. We performed several holes in the lower part of the succession. Samples representative of the magma-water interaction were collected in the field of the littoral explosion originated from the 2009 eruption of Pu’u’O at Waikupanaha, along the East Rift Zone. Downwind, at about 500–800 m from the cliff’s rim, we sampled Pele’s hair dispersed in the ash or hidden behind rocks or lava relict.

### Analytical methods

#### Microscopy observations and microanalysis

Fragments of Pele’s hair were separated from ash and their shape and external morphology were initially investigated under a binocular microscope and by a high-resolution optical microscope using reflected and transmitted polarized light. The inner structure of the fragments was observed on thin sections. Micromorphology and sub-micron textural details have been acquired using a Scanning Electron Microscope (SEM). SEM analyses have been performed with a JEOL JSM-5900 LV at the “W.M. Keck Cosmochemistry Laboratory” at the University of Hawaii at Manoa and a FEI/Philipsat SEM at the “Laboratorio di Microscopia Elettronica e Microanalisi” of the University of Calabria, Italy. We used secondary electrons detection to observe the surface topography of the samples (analytical conditions: 15 kV accelerating voltage and 26 mm working distance) and to image Pele’s hair surfaces, without excessive sample charging. Glass microanalysis was performed in the same laboratory with an Electron Microprobe JEOL JXA 8230 equipped with an EDS (Energy Dispersive X-ray Spectroscopy) microanalytical system JEOL-EX-94310FaL1Q-Silicon drift type. Analytical conditions were: 10 nA probe current, 15 keV accelerating voltage, 30 s live time, 4 µm defocused beam.

#### Synchrotron radiation X-ray computed microtomography measurements

The 3D study of the Pele’s hair morphology was performed by high-resolution SR-μCT^23^. Samples were imaged at the SYRMEP beamline of the Elettra synchrotron laboratory (Trieste, Italy)^21,29^. A filtered (filters: 1.5 mm Si + 0.025 mm Mo) polychromatic X-ray beam delivered by a bending magnet source illuminated the sample in transmission geometry. The sample-to-detector distance was set at 100 mm. For each experiment, either 1500 or 3000 projections were recorded during continuous rotation over 180 or 360 degrees, respectively, with an exposure time/projection of 1.5 s. Measurements were carried out with an effective pixel size of the detector set at 1.3 × 1.3 μm^2^ yielding a maximum field of view of about 2.7 × 2.7 mm^2^. The detector used was an air-cooled, 16 bit, sCMOS camera (Hamamatsu C11440-22C) with a 2048 × 2048 pixels chip coupled with a LSO:Tb scintillator screen (17 μm-thick on the top of a 170 μm-thick LSO substrate) and a high numerical aperture optics.

A total number of 28 samples collected at LOE, KI and HMM locations were imaged. Each experiment was performed scanning 4 to 6 Pele’s hairs at the same time (Supplementary Fig. S7). Since samples have a maximum length exceeding the field of view, scans were acquired at different overlapping positions along the hair axis to reconstruct the entire morphology (detailed acquisition parameters in Supplementary Table S4). The reconstruction of the 2D tomographic slices was done with the SYRMEP Tomo Project (STP) software suite^42,43^ by using the Filtered Back-Projection algorithm^44^ with ring artefacts removal^45^. Projection images acquired in phase-contrast mode display a mix of absorption contrast and edge-enhancement effects. To improve the reliability of the segmentation process and morphological analysis, and to fully exploit the potential of phase-contrast SR-μCT, we applied a single-distance phase-retrieval algorithm, based on the transport of intensity equation^46^, to the acquired sample projection images prior to the reconstruction. We set a value of 50 for the δ/β = γ parameter (ratio between the real and imaginary parts of the complex refractive index) after a manual optimization to preserve the microstructural features (small vesicles) visible in the reconstructed images without phase-retrieval. This algorithm removes, or at least reduces, the effect of the phase-contrast artifacts and the phase information retrieved provides a better contrast in the reconstructed images. There is generally a slight blurring of images, since acquiring conditions are not ideal (algorithms assume homogeneous monophase “phase objects” and perfectly monochromatic X-ray beam). These algorithms can be employed, with some caution, even on dense materials and with polychromatic light^35^.

### Image processing

Image visualization and quantification were performed using the Avizo® 9.3 commercial software. As several scans were collected to image the full length of each group of hair, stacks of reconstructed slices were first aligned and then merged using standard Avizo built in algorithms. Each hair was individually extracted, segmented and analyzed. Vesicle segmentation was done using a *marker-based watershed segmentation* module^47,48^. The complete procedure used in image processing is detailed in the Supplementary Information (Supplementary Methods, Supplementary Fig. S8 and Supplementary Table S5). From segmented vesicles we extracted quantitative information: vesicle total number, volume, length (*l*, the maximum of the Feret diameters), width (*Deq*, as equivalent circular diameter, see Supplementary Fig. S9), elongation (evaluated as *1−Deq / l*), vesicularity (volume of vesicles/total volume of the sample), VND, and the fraction of vesicles that have length equal to that of the sample. Quantified parameters (volumes, lengths, widths and elongations) are reported in the Supplementary data files. For each Pele’s hair (melt plus vesicle phases) we also computed volume (*V*_*hair*_), length (*L*_*hair*_) and thickness (*D*_*hair*_). Results are summarized in Table 1.

### Data analysis

In order to compute differences between the studied eruption types, we based the statistical analysis on the basis of three main parameters: (i) elongation and (ii) vesicle number density for SR-μCT datasets, (iii) Pele’s hair thickness measured by Scanning Electron Microscopy. Vesicle volumes, lengths and widths are much more affected by bias due to truncation at the borders of the images, so we can accurately quantify them for KI samples only, for which we extracted complete vesicles. Average vesicle size values for LOE and HMM (reported in Table 1) incorporate the truncation effects. To make a comparison between the eruption types by vesicle elongation, the data from each hair sample were grouped to increase the number of vesicles (HMM_T_, LOE_T_ and KI_T_, where the subscript T stands for total observation) and then checked for differences by means of standard statistical tests.

For statistical analysis we employed the non-parametric *Mann-Whitney-Wilcoxon U-test* to check differences between each pair of VND distributions for which we have few measured values and so the hypothesis of normality cannot be assumed. The tests verify the null hypothesis that data comes from continuous distributions with equal medians. A *One-way Anova* test with *Bonferroni* correction was used to check for significant differences in terms of vesicle elongation and hair thickness (comparing the population means). For both tests we used the associated functions of the *Statistics and Machine Learning* toolbox of Matlab®2018. A p-value of less than 0.05 was considered significant.

VSDs for KI samples are right-skewed with very long tails that can spread up over several orders of magnitude. Previous studies^20,49,50^ have shown that VSDs in volcanic rocks typically feature a logarithmic distribution, so a Log10-based conversion was applied in order to use linear distribution functions^20^. From transformed data it was possible to extract standard descriptive parameters, i.e. the mean (μ) and standard deviation (σ), that were back-transformed to original linear scale in order to obtain their geometric means (µ*, the median) and geometric standard deviations (σ*) that better characterize logarithmic distributions^51^. The back-transformations in natural units are: µ* = 10 ^µ^, σ* = 10^σ^. Considering the geometric moments, the confidence interval could be expressed as µ*∙/σ*. The notation ∙/ (times-divide) indicates that a confidence interval of 68.7% is expressed as [µ*/σ*, µ* ∙ σ*]^51^. This notation was shown to better determine useful intervals for logarithmic distributions of right-skewed data. Results of VSDs are reported in Table 2. Probability density functions of KI_T_’s vesicle volume, length and width are reported in Supplementary Fig. S5. The number of bins is selected accordingly to the Freedman-Diaconis rule.

## Supplementary information

Supplementary information

Dataset 1

Dataset 2

Dataset 3

Thickness map

## Data Availability

The dataset analyzed during the current study are available contacting the corresponding author on reasonable request.
