# Supplementary material for: First 3D imaging characterization of Pele’s hair from Kilauea volcano (Hawaii)
Source: Sci Rep. 2019 Feb 8;9:1711. doi: 10.1038/s41598-018-37983-9 (PMC6368622; doi:10.1038/s41598-018-37983-9)
Supplement: Supplementary file 1 — Supplementary information [file 41598_2018_37983_MOESM1_ESM.pdf]

Supplementary Information

First 3D imaging characterization of Pele's hair from Kilauea volcano (Hawaii)

Cannata C.B. ; De Rosa, R.; Donato, P.; Donato, S.; Lanzafame, G.; Mancini, L.; Houghton, B. F.

Supplementary Methods

Stacks of reconstructed slice were firstly converted to 8-bit values (due to the large size of the data), then registered and merged using built in functions of Avizo (*Register Image* and *Merge*). After that, each hair was selected and extracted using the *Extract Subvolume* module for further preprocessing and segmentation. Due to the slight blurring introduced by the phase-retrieval processing, an *Unsharp masking* module was applied to sharpen edges, particularly thin films between adjacent vesicles, without increasing the noise. This filter generates a Gaussian smoothing of an image that is then subtracted away from the original image to detect the presence of edges. The amount of sharpening is controlled building a mask, from the gradient image, and applying it only to edges above a certain threshold size (*Radius* in Supplementary Table S5). The *auto-thresholding* module was applied to select only high intensity voxels (melt phase of the hair) on the basis of their gray levels using the Otsu criterion [1]. Then, a series of morphological operators were used in order to close the vesicles, obtaining the total volume of each hair. These Volumes of Interest (VOIs) were used as a mask to further segment the hosted vesicles with an *interactive* (manual) thresholding module. Since vesicles of the samples are sometimes separated by very thin films, a *marker-based watershed segmentation* [2] module was applied using markers as prior knowledge. This procedure requires to calculate a distance map [2] (a chessboard-like distance map in our case) to assign to each background pixel the distance from the foreground. This gray image is interpreted as landscape height field that controls the flood process and the location of watershed separation. Markers are selected applying an *H-maxima* transform [2] to the distance map suppressing all the maxima whose depth is below or equal to a given threshold given as input parameter. Finally, the watershed segmentation was started using markers as seed points. The obtained watershed lines were subtracted from the binary image of segmented vesicles that are then labeled and individually analyzed. The main steps of image segmentation are shown in Supplementary Fig. S8. Evident vesicle breaks due to segmentation faults are corrected by means of linear interpolation using the *Segmentation Editor* of Avizo. For KI samples, due to the large amount of vesicles, a *borderkill* module was used to suppress labels intersecting the borders of the sample. For KI samples we computed also: (i) sphericity (s, defined as $s = \pi^{1/3}(6V)^{2/3}/A$, where V and A stand for volume and 3D area of the vesicle, respectively) and (ii) the diameter of the equivalent sphere obtained from the volume of the vesicle ($d = 2*(0.75*V/\pi)^{1/3}$). The quantification of vesicle parameters was performed using the *label analysis module* of Avizo. For KI3 and KI6 samples we evaluated the local vesicle thickness using the *Thickness Map* module. It computes the diameter of the largest ball containing the voxel and entirely inscribed in the vesicle [3].

References

[1] Otsu, N. A thresholding selection method from grayscale histogram. *IEEE Trans. Syst. Man Cybern*. **9**, 62-66 (1979).
[2] Soille, P. Morphological Image Analysis: Principles and Applications. (2nd ed. Springer), Berlin/Heidelberg, Germany (2004).

[3] Hildebrand, T. & Ruegsegger, P. A new method for the model-independent assessment of thickness in three-dimensional images. *J.Microscopy*, **185**:1 (1997).

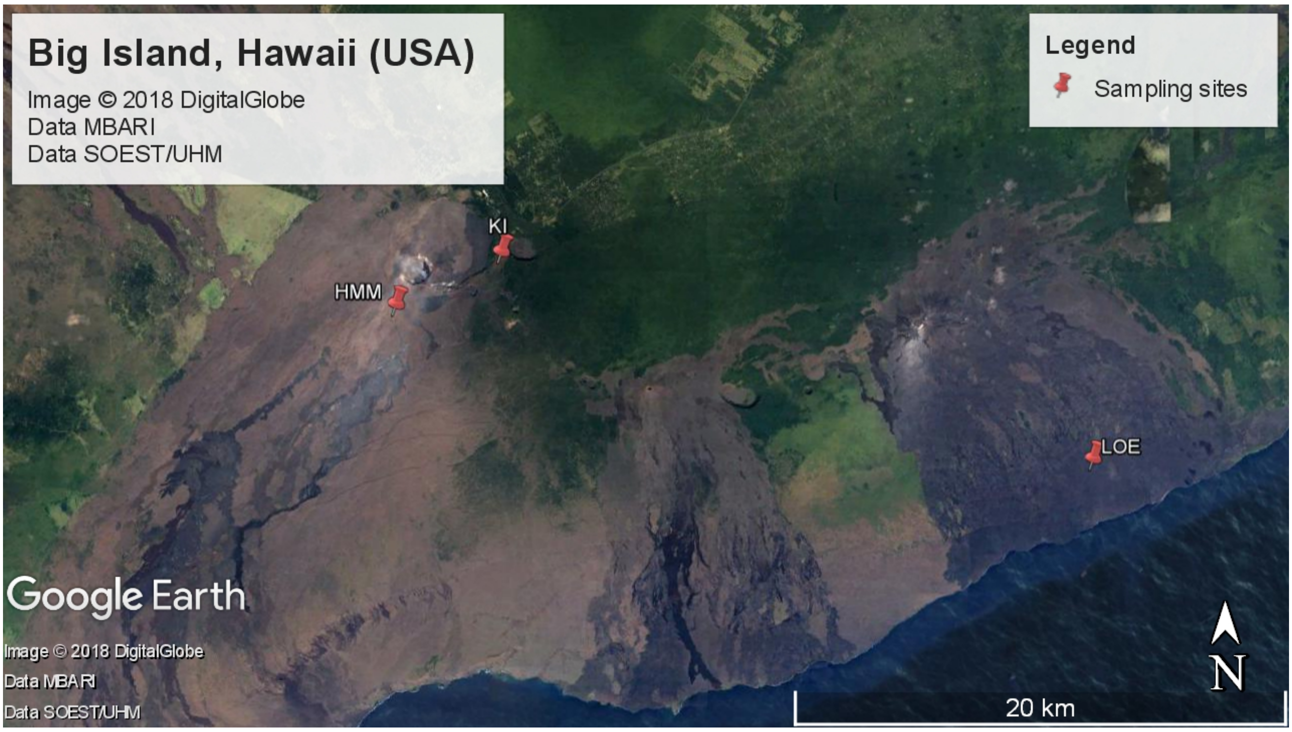

Supplementary Figure S1: Site of sampling for HMM, LOE, KI samples (image from Google Earth: Image © 2018 DigitalGlobe, Data MBARI, Data SOEST/UHM).

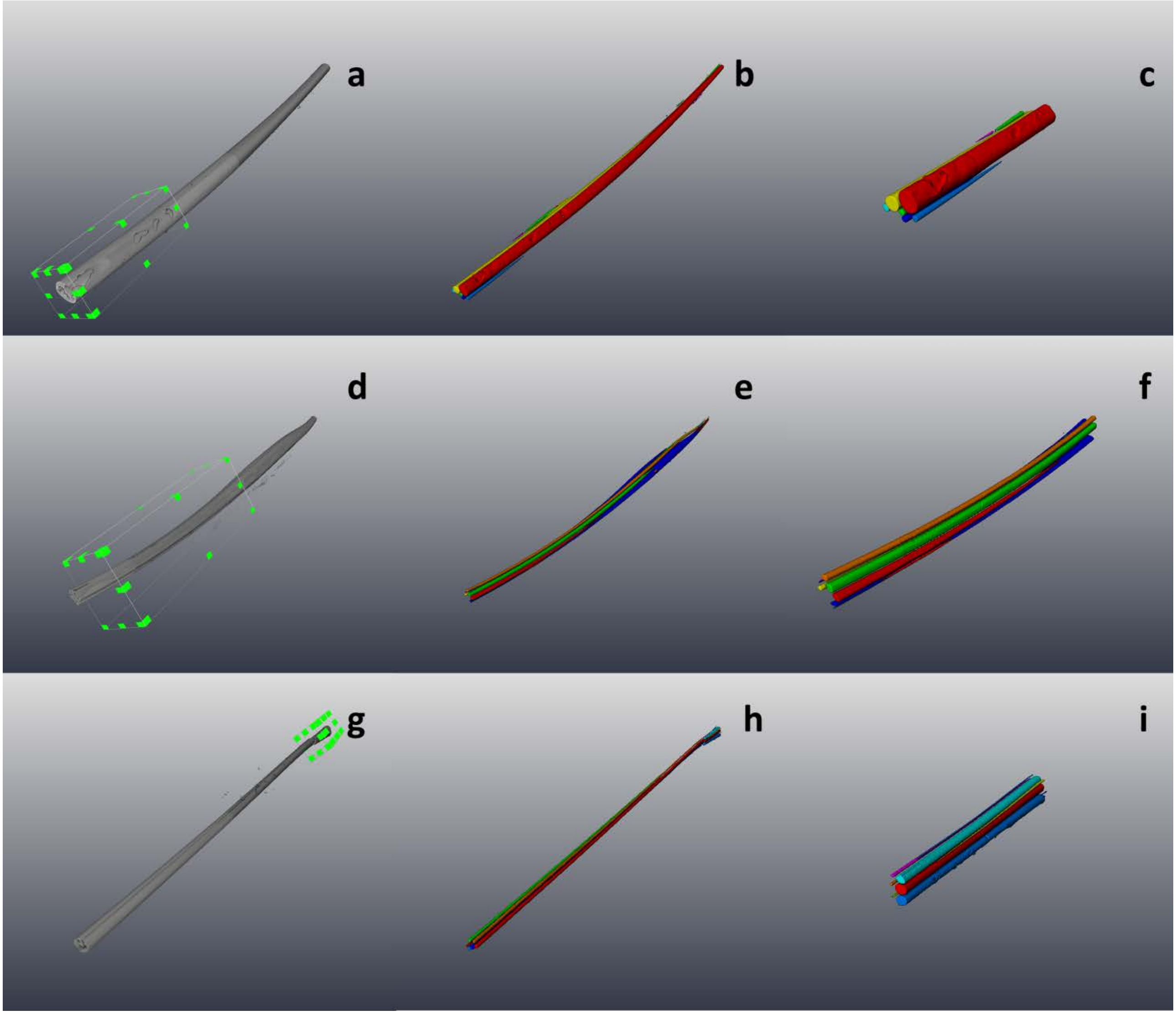

Supplementary Figure S2: (a,d,g) 3D rendering the imaged HMM Pele's hairs in grayscale for sample 1, 2 and 3; (b,e,h) Corresponding labeled vesicles, displayed with an RGB colormap; (c,f,i). Enlargement of the labeled vesicles, selected from a region of interest (marked with a box) on the grayscale 3D images.

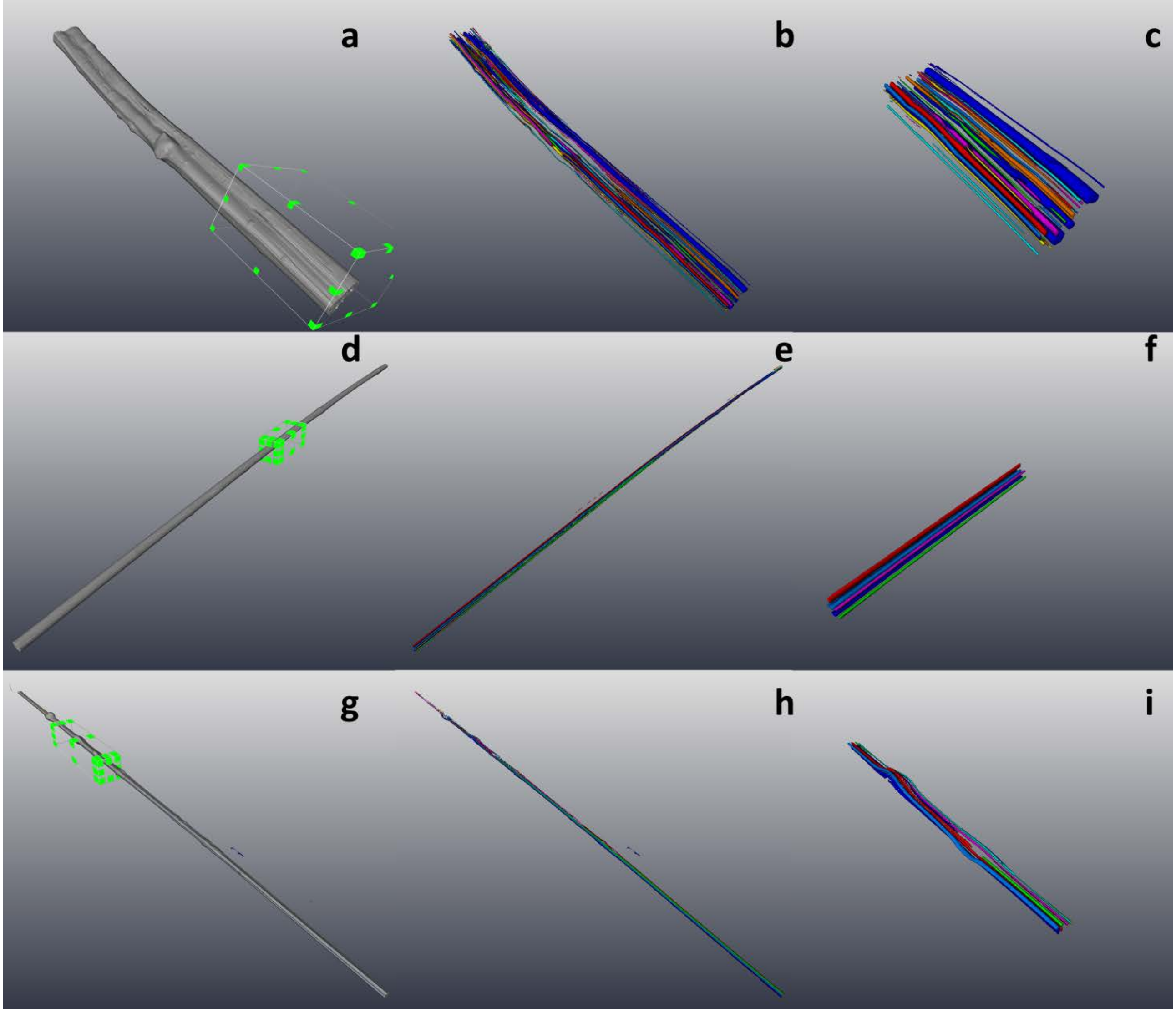

Supplementary Figure S3: (a,d,g) 3D rendering the imaged LOE Pele's hairs in grayscale for sample 1, 3 and 8; (b,e,h) Corresponding labeled vesicles, displayed with an RGB colormap; (c,f,i) Enlargement of the labeled vesicles, selected from a region of interest (marked with a box) on the grayscale 3D images.

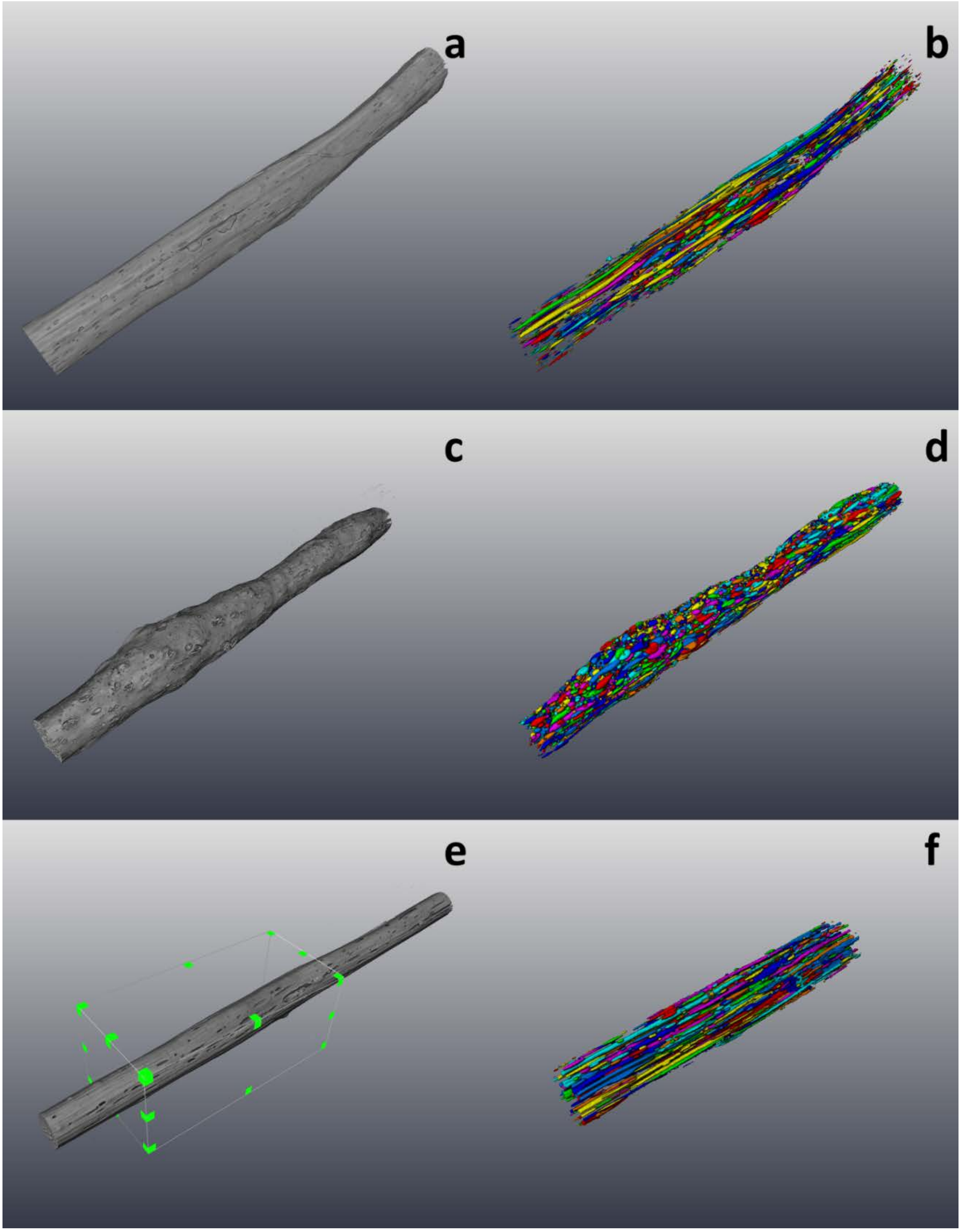

Supplementary Figure S4: (a,d,g) 3D rendering the imaged KI Pele's hairs in grayscale for sample 1, 6 and 8; (b,d,f) Corresponding labeled vesicles, displayed with an RGB colormap.

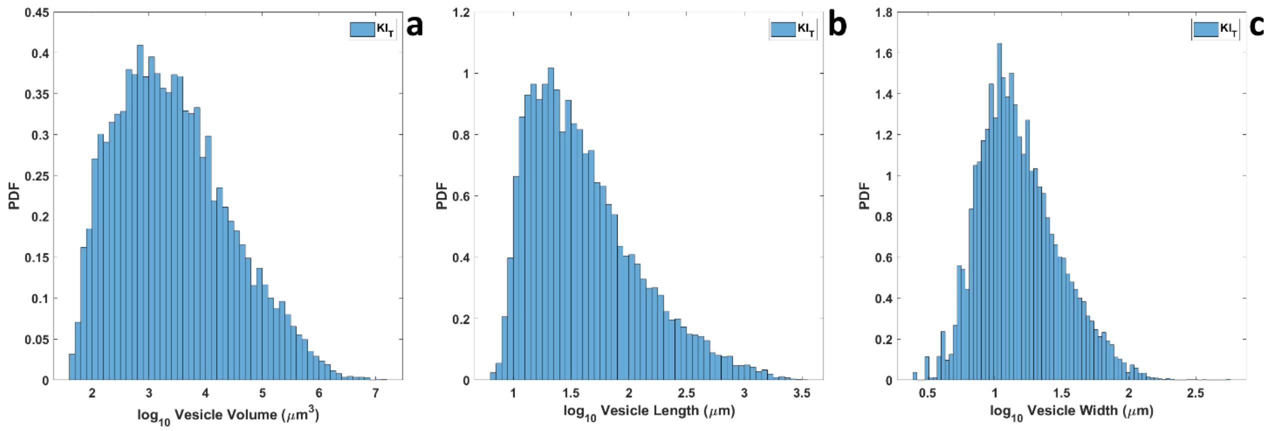

Supplementary Figure S5: Log10 VSDs for all vesicles collected from KI samples. (From left to right) Probability density function of vesicle volume, length and width (from the equivalent circular diameter Deq). The logarithmic transform enable a quasi-normalization of the distribution. From average and standard deviation of Log-10 distribution we can back-transform to obtain geometric moment of the data in natural units: i.e. the median and geometric standard deviation. These two quantities enable to define confidence interval reported in Table 2 of the manuscript.

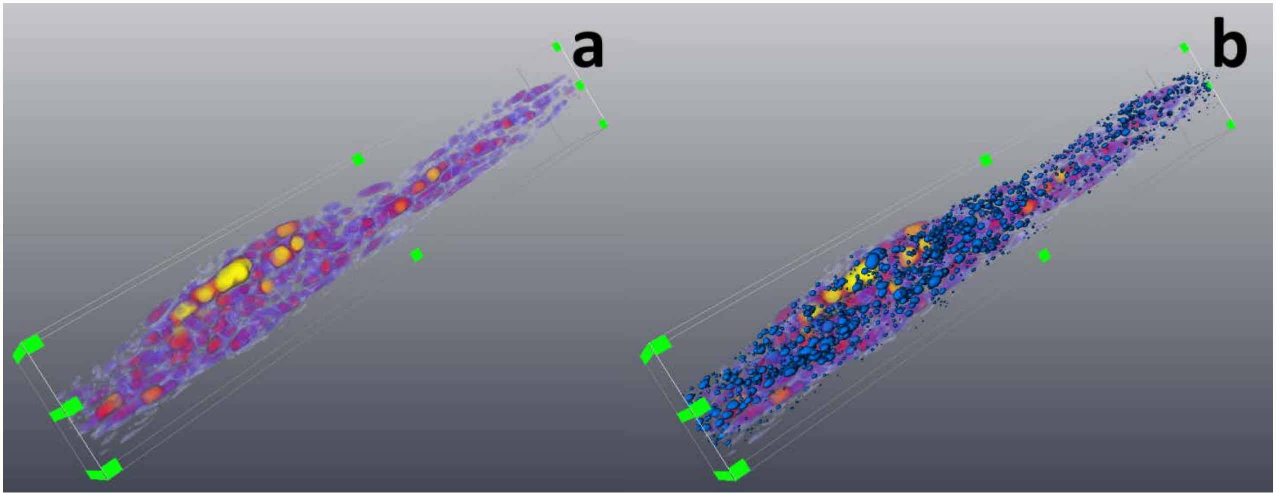

Supplementary Figure S6: (a) 3D rendering showing a sagittal cut of the vesicles thickness map (for KI sample no. 6) displayed using a color code: yellow colors correspond to the higher vesicle thickness; (b) 3D rendering of vesicles (blue objects) having sphericity > 0.9 overlayed to the thickness map. Spherical vesicles of large size are mostly located in the swelled section of the hair.

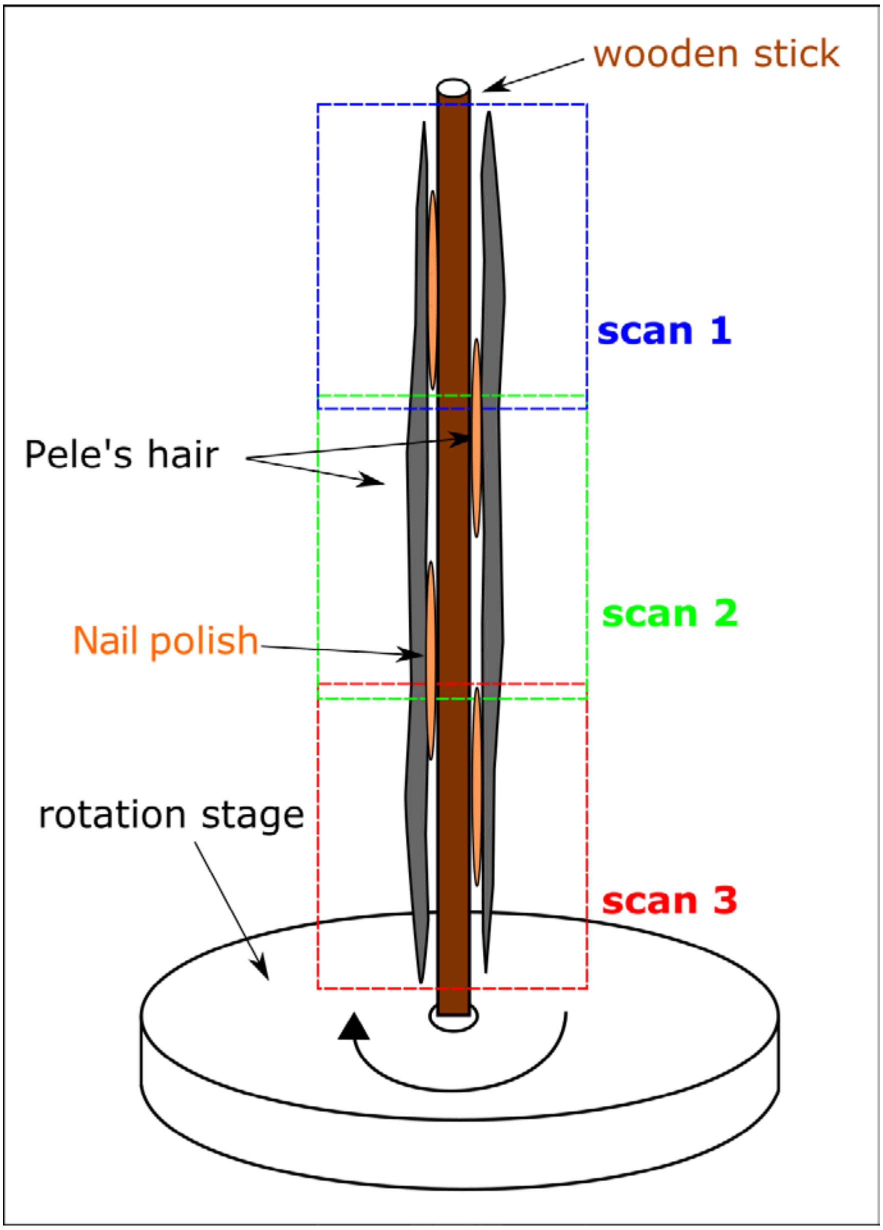

Supplementary Figure S7: Schematic example of the multiple scans analysis. Typically, 4 to 6 Pele's hairs were glued with nail polish to a wooden stick and mounted with their elongation axis parallel to the rotational axis of the rotation stage. Multiple scans were performed with an overlap of ~ 0.3 mm between two consecutive scans.

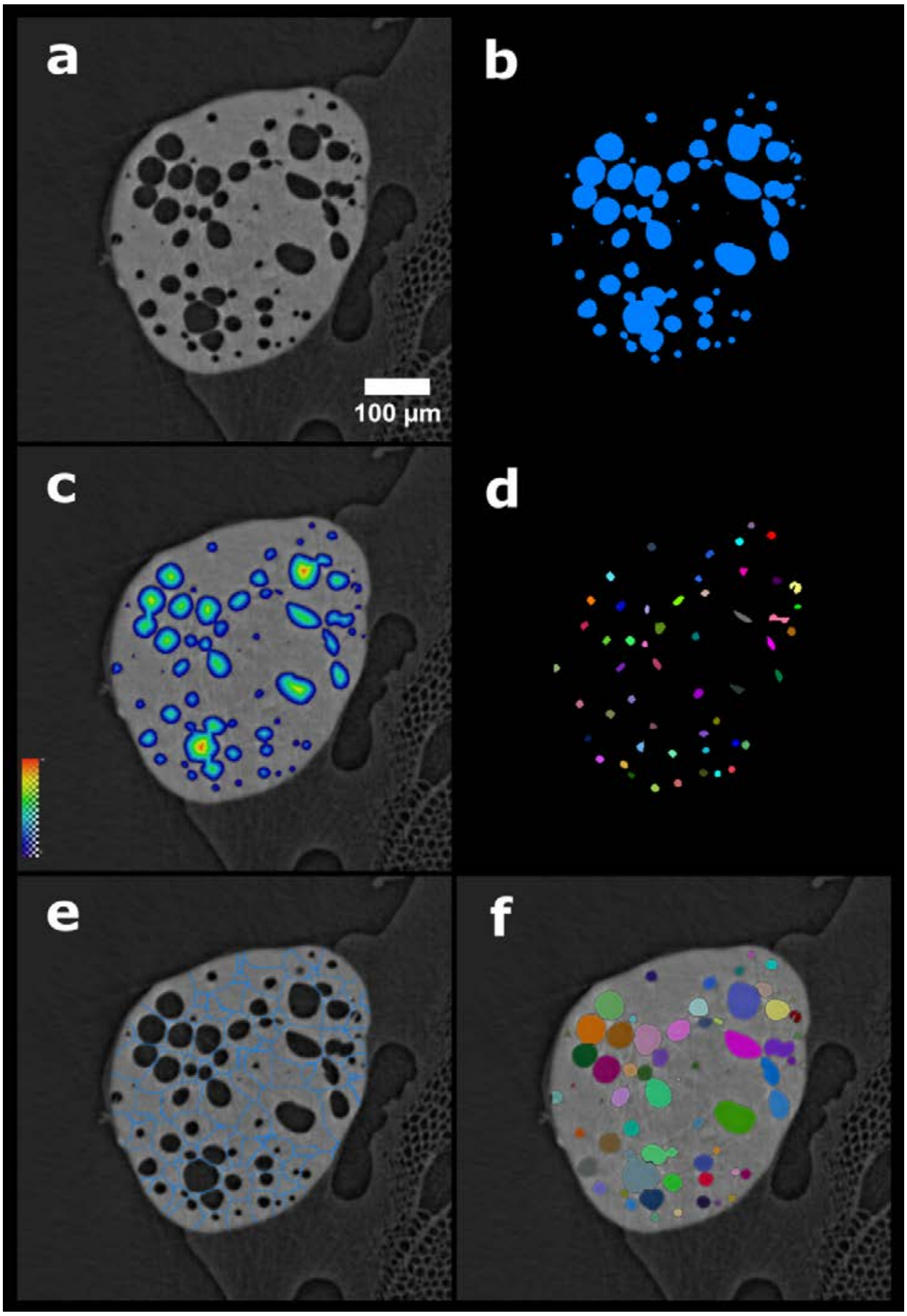

Supplementary Figure S8: Main steps of vesicle segmentation procedure. a) Axial reconstructed slice sharpened to enhance edge contrast; b) Segmented vesicles using the manual thresholding; c) Chessboard distance map overlaid on the grayscale slice, color gradients represent the Chessboard distance from the borders (red color corresponds to the higher values); d) labeled markers used for watershed segmentation computed from the H-Maxima module; e) Computed watershed lines that show the effective vesicle separation; f) Separated vesicles, each color indicates a different label. Isotropic pixel size of 1.3x1.3 μm^2.

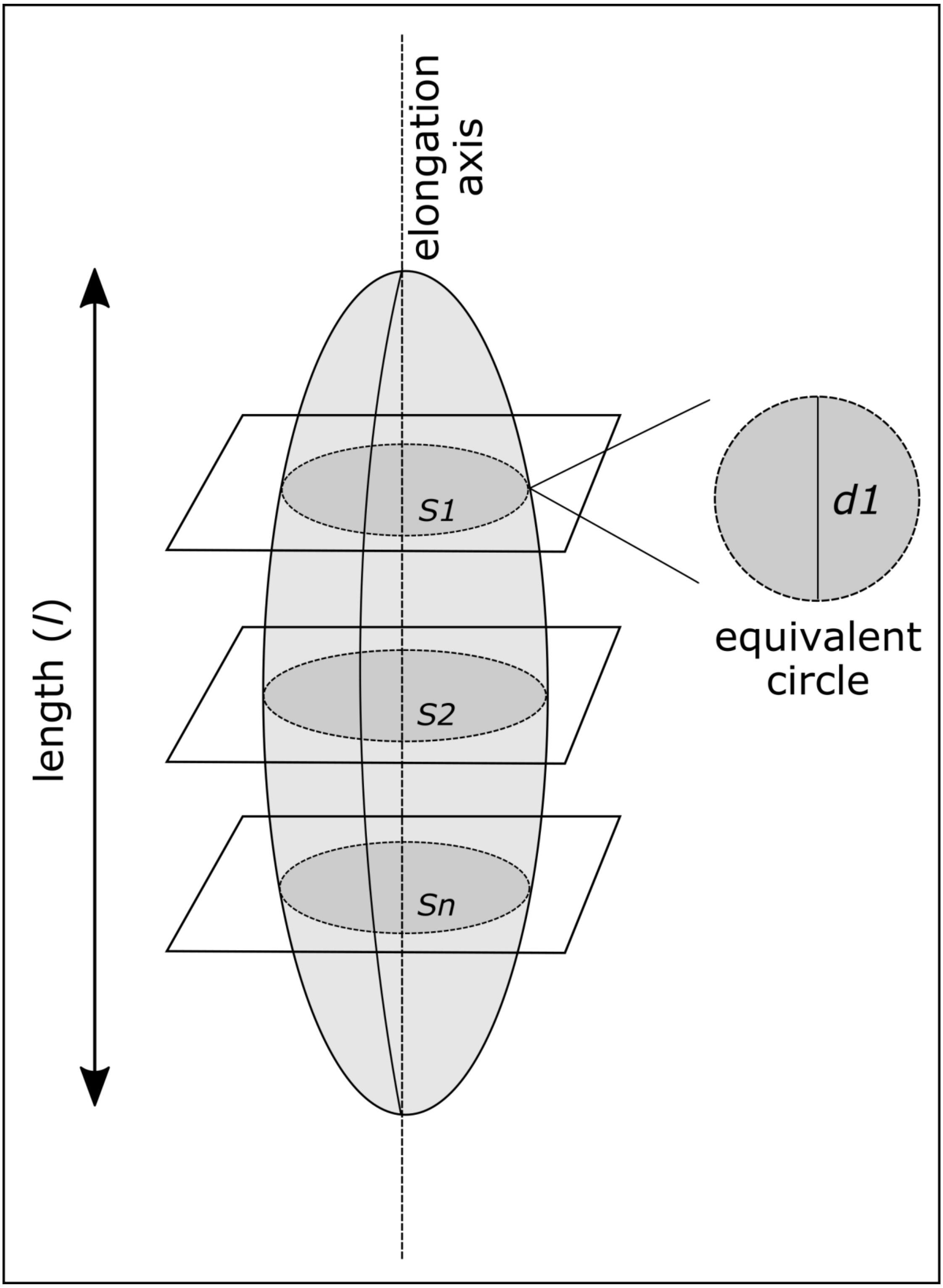

Supplementary Figure S9: Measure of the vesicle width as equivalent circular diameter (*Deq*), computed as the average value of the n diameters of the equal area circles (*d1*) of the n surfaces orthogonal to the vesicle elongation axis.

KI				LOE				HMM (N=71)			
#	Thickness (μm)	#	Thickness (μm)	#	Thickness (μm)	#	Thickness (μm)	#	Thickness (μm)	#	Thickness (μm)
**1**	552	**41**	201	**1**	238	**41**	181	**1**	382	**41**	119
**2**	902	**42**	152	**2**	177	**42**	168	**2**	155	**42**	85
**3**	470	**43**	198	**3**	346	**43**	305	**3**	58	**43**	59
**4**	502	**44**	189	**4**	61	**44**	127	**4**	70	**44**	90
**5**	666	**45**	415	**5**	75	**45**	85	**5**	86	**45**	58
**6**	234	**46**	205	**6**	167	**46**	228	**6**	421	**46**	76
**7**	183	**47**	552	**7**	285	**47**	161	**7**	60	**47**	74
**8**	324	**48**	206	**8**	155	**48**	461	**8**	38	**48**	32
**9**	450	**49**	462	**9**	391	**49**	89	**9**	65	**49**	42
**10**	306	**50**	99	**10**	313	**50**	279	**10**	46	**50**	49
**11**	278	**51**	226	**11**	13	**51**	473	**11**	77	**51**	62
**12**	361	**52**	108	**12**	36	**52**	193	**12**	52	**52**	45
**13**	243	**53**	127	**13**	43	**53**	171	**13**	35	**53**	42
**14**	33	**54**	244	**14**	536	**54**	149	**14**	31	**54**	77
**15**	161	**55**	167	**15**	227	**55**	149	**15**	29	**55**	59
**16**	142	**56**	391	**16**	49	**56**	122	**16**	77	**56**	73
**17**	219	**57**	270	**17**	79	**57**	139	**17**	35	**57**	54
**18**	265	**58**	244	**18**	175	**58**	97	**18**	62	**58**	64
**19**	365	**59**	505	**19**	391	**59**	135	**19**	64	**59**	40
**20**	293	**60**	134	**20**	357	**60**	105	**20**	31	**60**	51
**21**	236	**61**	254	**21**	147	**61**	74	**21**	53	**61**	86
**22**	234	**62**	206	**22**	205	**62**	122	**22**	59	**62**	25
**23**	115	**63**	250	**23**	343	**63**	106	**23**	96	**63**	35
**24**	149	**64**	170	**24**	263	**64**	113	**24**	57	**64**	76
**25**	109	**65**	123	**25**	257	**65**	100	**25**	67	**65**	68
**26**	200	**66**	144	**26**	87	**66**	132	**26**	64	**66**	129
**27**	88	**67**	151	**27**	496	**67**	141	**27**	73	**67**	68
**28**	287	**68**	132	**28**	78	**68**	302	**28**	109	**68**	79
**29**	601	**69**	159	**29**	191	**69**	142	**29**	75	**69**	52
**30**	314	**70**	336	**30**	109	**70**	269	**30**	24	**70**	105
**31**	115	**71**	340	**31**	156	**71**	105	**31**	24	**71**	61
**32**	293	**72**	115	**32**	202	**72**	184	**32**	74		
**33**	271	**73**	242	**33**	92	**73**	135	**33**	59		
**34**	158	**74**	245	**34**	181	**74**	77	**34**	89		
**35**	469	**75**	165	**35**	178	**75**	205	**35**	59		
**36**	544	**76**	304	**36**	120	**76**	40	**36**	119		
**37**	163	**77**	455	**37**	291	**77**	535	**37**	97		
**38**	196	**78**	432	**38**	292	**78**	135	**38**	59		
**39**	200			**39**	179	**79**	173	**39**	33		
**40**	135			**40**	151	**80**	97	**40**	67		

KI (N=78)		LOE (N=80)		HMM (N=71)	
**Mean**	**271**	**Mean**	**189**	**Mean**	**74**
**σ**	**156**	**σ**	**118**	**σ**	**62**
**Mode**	**206**	**Mode**	**122**	**Mode**	**59**
**Median**	**234**	**Median**	**158**	**Median**	**62**

Supplementary Table S1: Pele's hair thickness measured under the Scanning Electron Microscope. As the shape of the Pele’s hair is not constant, the measure has been taken where no “knots” (thickening) or visible thinning of the Pele’s hair occur. The main statistical parameters for each group of samples is also reported.

	Sample	SiO_2	TiO_2	Al_2O_3	FeO_T	MnO	MgO	CaO	Na_2O	K_2O	Total
**HMM**	HMM_01	52.54	2.47	13.86	10.14	b.d.l.	7.52	10.67	2.35	0.44	100
	HMM_02	52.49	2.67	13.82	9.48	b.d.l.	7.54	10.99	2.3	0.71	100
	HMM_03	52.08	2.25	13.82	10.7	b.d.l.	7.68	10.71	2.36	0.41	100
	HMM_04	52.02	2.67	13.66	10.55	b.d.l.	7.45	10.74	2.47	0.44	100
	HMM_05	52.67	2.2	13.48	10.24	b.d.l.	7.5	10.95	2.47	0.5	100
	HMM_06	51.96	2.36	13.44	10.53	b.d.l.	7.77	11.23	2.38	0.33	100
	HMM_07	52.26	2.34	13.31	10.89	b.d.l.	7.53	11.01	2.24	0.42	100
	HMM_08	51.81	2.45	13.73	10.42	0.63	7.63	10.66	2.22	0.44	100
	HMM_09	52.2	2.38	13.55	10.8	b.d.l.	7.53	10.7	2.33	0.49	100
	HMM_10	52.17	2.44	13.92	10.17	0	7.67	10.88	2.25	0.49	100
	**Mean**	**52.22**	**2.42**	**13.66**	**10.39**	**0.32**	**7.58**	**10.85**	**2.34**	**0.47**	**100**
	σ	*0.27*	*0.16*	*0.21*	*0.41*	*0.45*	*0.10*	*0.19*	*0.09*	*0.10*	
**KI**	KI_01	50.3	2.38	12.55	10.82	b.d.l.	9.77	11.4	2.29	0.49	100
	KI_02	50.41	2.19	12.44	10.6	b.d.l.	10.55	11.11	2.1	0.6	100
	KI_05	50.15	2.69	12.23	10.84	b.d.l.	10.27	11.05	2.19	0.57	100
	KI_06	50.36	2.63	12.1	11.05	b.d.l.	10.1	11.15	2.14	0.47	100
	KI_07	50.15	2.37	12.33	11.5	b.d.l.	10.08	11.1	2.01	0.46	100
	KI_08	50.36	2.59	12.29	11.15	b.d.l.	9.54	11.49	2.05	0.53	100
	KI_09	49.79	2.6	12.37	10.89	b.d.l.	10.57	11.04	2.25	0.5	100
	KI_10	49.98	2.3	12.4	11.33	b.d.l.	10.7	10.81	1.9	0.59	100
	KI_11	49.81	2.63	12.19	10.5	b.d.l.	10.23	11.35	2.52	0.78	100
	KI_12	49.89	2.44	12.3	11.24	b.d.l.	10.06	11.28	2.17	0.62	100
	**Mean**	**50.12**	**2.48**	**12.32**	**10.99**	**_**	**10.19**	**11.18**	**2.16**	**0.56**	**100**
	σ	*0.24*	*0.17*	*0.13*	*0.32*	_	*0.36*	*0.20*	*0.17*	*0.10*	
**LOE**	LOE_01	52.6	2.5	13.66	10.58	b.d.l.	7.03	10.84	2.34	0.45	100
	LOE_02	52.08	2.64	13.77	10.79	b.d.l.	7.03	10.9	2.38	0.41	100
	LOE_03	52.65	2.34	13.92	10.48	b.d.l.	6.95	10.77	2.41	0.48	100
	LOE_04	52.27	2.5	14.17	10.47	b.d.l.	6.87	10.88	2.45	0.38	100
	LOE_05	52.02	2.45	13.76	10.8	b.d.l.	7.02	10.88	2.67	0.42	100
	LOE_06	52.24	2.3	14.12	10.59	b.d.l.	7.05	11.03	2.38	0.29	100
	LOE_07	52.25	2.38	13.87	10.8	b.d.l.	6.98	10.73	2.48	0.5	100
	LOE_08	52.34	2.55	13.88	10.43	b.d.l.	7.03	10.94	2.4	0.43	100
	LOE_09	52.21	2.45	13.97	10.56	b.d.l.	6.83	11.21	2.37	0.41	100
	LOE_10	52.31	2.47	13.73	10.56	b.d.l.	6.86	11.2	2.48	0.37	100
	**Mean**	**52.30**	**2.46**	**13.89**	**10.61**	**_**	**6.97**	**10.94**	**2.44**	**0.41**	**100**
	σ	*0.20*	*0.10*	*0.17*	*0.14*	_	*0.08*	*0.16*	*0.09*	*0.06*	

Supplementary Table S2: Pele's hair glass composition determined by Energy Dispersive X-ray Spectroscopy microanalysis. Details on the acquisition parameters are in the 4.2.1 subsection.

H_2O: 0.5 wt.%		
**Sample**	**Viscosity η log (Pa s)**	**T Liquidus (°C)**
**HMM**	1.67	1187
**KI**	1.19	1211
**LOE**	1.62	1201

Supplementary Table S3: Calculated viscosity and liquidus temperature on the basis of chemical composition. Details on the calculation are in the text.

Group name	Sample to detector distance (mm)	# of samples imaged at the same time	# of vertical scans per group	# of projection s per scan	Exposure time per projection (s)	Total angular range (°)
HMM	100	6	3	1500	1.5	180
LOE	100	4	3	1500	1.5	180
K1	100	5	2	1500	1.5	180
K1_BIS	100	4	2	3000	1.5	360
LOE_BIS	100	4	4	1500	1.5	180
HMM_LONG	100	5	5	1500	1.5	180

Supplementary Table S4: Acquisition parameters of the synchrotron radiation computed microtomography (XCT) experiment. The second column report the sample-to detector distance used to perform perform phase-contrast enhanced XCT. We collected different samples at the same time (third column) and covered the full length by consecutive vertical scan (fourth column) with a small overlap to enable the merging of image stacks. For each vertical step, a fixed number of projections (fifth column) were collected using fidex exposure time of 1.5 s and pixel size of 1.3x1.3 μm^2. Every group of samples were collected over 180° except the group KI_BIS which required an extended field-of-view acquisition over 360°. In HMM group were imaged samples from 1 to 6B, in HMM_LONG samples from 7 to 11 (see sample list in Tab.1 of the main text). In LOE group were imaged samples from 1 to 4, while in LOE_BIS samples from 5 to 8. Finally, in group KI were imaged samples from 1 to 5, and in KI_BIS samples from 6 to 9.

	Unsharp masking module			Auto thresholding (Otsu criterion)	Interactive (manual) thresholding	H-Maxima transform
	Radius	*Edge Contrast*	*Brightness threshold*	*8 bit gray value*	*8 bit gray value*	*Contrast size*
LOE SAMPLES						
LOE_1	4	0.5	150	146	142	4
LOE_2	4	0.5	150	146	140	4
LOE_3	2	0.5	150	146	131	3
LOE_4	2	0.5	150	146	127	3
LOE_5	3	0.5	150	146	138	3
LOE_6	2	0.5	150	146	135	2
LOE_7	3	0.5	150	146	137	3
LOE_8	2	0.5	150	146	130	3
KI SAMPLES						
KI_1	4	0.5	140	135	125	4
KI_2	3	0.5	140	135	130	4
KI_3	4	0.5	140	135	123	5
KI_4	4	0.5	140	135	125	5
KI_5	3	0.5	140	135	127	4
KI_6	4	0.5	140	135	133	4
KI_7	4	0.5	140	135	126	3
KI_8	4	0.5	140	135	133	4
KI_9	4	0.5	140	135	137	4
HMM SAMPLES						
HMM_1	4	0.5	150	145	138	5
HMM_2	4	0.5	150	145	136	4
HMM_3_A	4	0.5	150	145	140	4
HMM_3_B	4	0.5	150	145	140	5
HMM_4	2	0.5	150	145	137	2
HMM_5	4	0.5	150	145	140	4
HMM_6_A	4	0.5	150	145	141	5
HMM_6_B	2	0.5	150	145	138	3
HMM_7	4	0.5	150	148	142	3
HMM_8	2	0.5	150	148	144	2
HMM_9	2	0.5	150	148	143	2
HMM_10	2	0.5	150	148	144	3
HMM_11	2	0.5	150	148	144	2

Supplementary Table S5: Main parameters for image segmentation for each studied sample. Unsharp Masking module requires three parameters: (i) *Radius* affect the size of the edges to be enhanced, only edges above this value are considered in the process (fine details need a smaller radius); (ii) *Edge Contrast* defines the amount of contrast added at the processed edges; (iii) Brightness threshold set the minimum gray value of the image to be considered when the gradient image has to be calculated. *Auto thresholding* column report the 8-bit gray value, based on the Otsu criterion, to select only voxels of the melt phase of

the hair. *Interactive thresholding* column reports the manually selected gray values for vesicle segmentation. *H-maxima* column report the threshold applied to the distance map to suppress all maxima lower or equal to this value.

Supplementary Table S6: Quantitative results of the analysis of vesicles done in Avizo for each samples of HMM Pele's hair ("HMM_vesicle_results" excel file in Supplementary data). Sheet names indicate the corresponding studied sample. The first column is the volume of the vesicle in μm^3. The second column report the vesicle length (maximum of the Feret diameters) in µm.
Equivalent diameter is reported in the third column and expressed in µm. The fourth column is the vesicle elongation.

Supplementary Table S7: Quantitative results of the vesicle analysis done in Avizo for each samples of LOE Pele's hair ("LOE_vesicle_results" excel file in Supplementary data). Sheet names indicate the corresponding studied sample. The first column is the volume of the vesicle in μm^3. The second column report the vesicle length (maximum of the Feret diameters) in µm. Equivalent diameter is reported in the third column and expressed in µm. The fourth column is the vesicle elongation.

Supplementary Table S8: Quantitative results of the vesicle analysis done in Avizo for each samples of KI Pele's hair ("KI_vesicle_results" excel file in Supplementary data). Sheet names indicate the corresponding studied sample. The first column is the volume of the vesicle in μm^3. The second column report the vesicle length (maximum of the Feret diameters) in µm. Equivalent diameter is reported in the third column and expressed in µm. The fourth column is the vesicle elongation. Vesicle sphericity is reported in the fifth column.
